# Efficient Semi-Transparent Wide-Bandgap Perovskite Solar Cells Enabled by Pure-Chloride 2D-Perovskite Passivation

**DOI:** 10.1007/s40820-023-01090-w

**Published:** 2023-04-30

**Authors:** Liu Yang, Yongbin Jin, Zheng Fang, Jinyan Zhang, Ziang Nan, Lingfang Zheng, Huihu Zhuang, Qinghua Zeng, Kaikai Liu, Bingru Deng, Huiping Feng, Yujie Luo, Chengbo Tian, Changcai Cui, Liqiang Xie, Xipeng Xu, Zhanhua Wei

**Affiliations:** 1 Xiamen Key Laboratory of Optoelectronic Materials and Advanced Manufacturing, Institute of Luminescent Materials and Information Displays, College of Materials Science and Engineering, Huaqiao University, Xiamen, 361021 People’s Republic of China; 2 MOE Engineering Research Center for Brittle Materials Machining, Institute of Manufacturing Engineering, College of Mechanical Engineering and Automation, Huaqiao University, Xiamen, 361021 People’s Republic of China; 3 Gold Stone (Fujian) Energy Company Limited, Quanzhou, 362005 People’s Republic of China; 4 Collaborative Innovation Center of Chemistry for Energy Materials, Department of Chemistry, College of Chemistry and Chemical Engineering, Xiamen University, Xiamen, 361005 People’s Republic of China

**Keywords:** Wide-bandgap perovskite solar cells, Transparent back electrodes, Defect passivation, Bulky cations

## Abstract

Wide-bandgap (WBG) perovskite solar cells suffer from severe non-radiative recombination and exhibit relatively large open-circuit voltage (*V*_OC_) deficits, limiting their photovoltaic performance. Here, we address these issues by in-situ forming a well-defined 2D perovskite (PMA)_2_PbCl_4_ (phenmethylammonium is referred to as PMA) passivation layer on top of the WBG active layer. The 2D layer with highly pure dimensionality and halide components is realized by intentionally tailoring the side-chain substituent at the aryl ring of the post-treatment reagent. First-principle calculation and single-crystal X-ray diffraction results reveal that weak intermolecular interactions between bulky PMA cations and relatively low cation-halide hydrogen bonding strength are crucial in forming the well-defined 2D phase. The (PMA)_2_PbCl_4_ forms improved type-I energy level alignment with the WBG perovskite, reducing the electron recombination at the perovskite/hole-transport-layer interface. Applying this strategy in fabricating semi-transparent WBG perovskite solar cells (indium tin oxide as the back electrode), the *V*_OC_ deficits can be reduced to 0.49 V, comparable with the reported state-of-the-art WBG perovskite solar cells using metal electrodes. Consequently, we obtain hysteresis-free 18.60%-efficient WBG perovskite solar cells with a high *V*_OC_ of 1.23 V.

**Graphical Abstract:**

**Supplementary Information:**

The online version contains supplementary material available at 10.1007/s40820-023-01090-w.

## Highlights

- Three bulky cation chlorides (PMACl, PEACl and NMACl) are used to modify the perovskite surface and form pure-anion 2D (PMA)_2_PbCl_4_, mixed-anion 2D (PEA)_2_Pb(I_*x*_Cl_4-*x*_), and non-2D NMAI passivation layers, respectively.
- Intermolecular interactions between the bulky cations and the strength of cation-halide hydrogen bonds are critical to forming the three distinct passivation layers.
- Semi-transparent wide-bandgap perovskite solar cells (WBG-PSCs) with ITO as the back electrode show hysteresis-free PCE of 18.60% and *V*_OC_ deficit of 0.49 V.

**Supplementary Information:**

The online version contains supplementary material available at 10.1007/s40820-023-01090-w.

## Graphical abstract

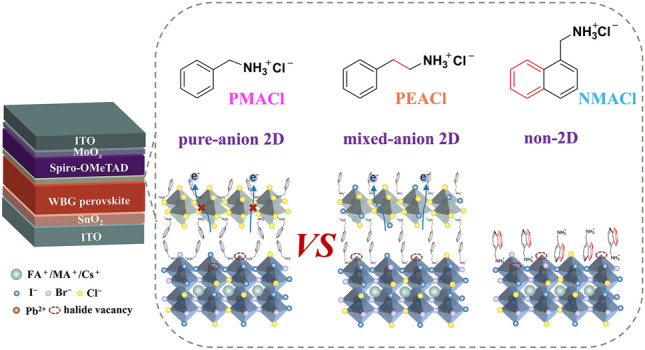

## Introduction

Perovskite solar cells (PSCs) have made unprecedented development in the past few years with certified power conversion efficiency (PCE) soaring from 3.8% in 2009 to the current 25.7% [1–6]. Despite the rapid improvement, the PCE of single-junction PSCs is fundamentally limited by the Shockley-Queisser (S-Q) limit [7]. One of the most promising strategies for surpassing the S-Q limit is to construct perovskite-based tandem solar cells by stacking a wide-bandgap (WBG) perovskite top cell [8] on a narrow-bandgap bottom cell (e.g., crystalline silicon (c-Si) [9–12], copper indium gallium selenide (CIGS) [13, 14], or lead (Pb)-tin (Sn) mixed perovskite [15–17]) to reduce the thermalization losses that originated from hot-carriers relaxation. Nowadays, the highest PCE of perovskite/silicon and perovskite/perovskite tandem solar cells has reached 32.5% and 29.0% [1, 18], respectively, which makes this technology greatly promising in the future photovoltaic market. However, the PCEs of perovskite-based tandem solar cells are still greatly lower than the theoretical limit of over 40% [19]. Among them, wide-bandgap perovskite solar cells are an important component of such tandem, but their performance is limited by large open-circuit voltage (*V*_OC_) deficits. At present, increasing the bromide content in the X site is a common strategy for preparing WBG perovskite. So WBG perovskite will face more unique challenges than the narrow-bandgap perovskite, such as smaller grains, more grain boundaries, and more serious photo-induced phase segregation [20]. Moreover, the energy levels of WBG perovskite may mismatch with the carrier transport layer that is commonly used in high-efficient narrow-bandgap devices. Therefore, it can lead to large *V*_OC_ deficits.

In the past few years, many efforts have been devoted to reducing the *V*_OC_ deficits of WBG-PSCs. Strategies such as compositional engineering, adding additives, crystallization control, and interface passivation were adopted to increase the film quality and improve the energy level arrangement of WBG-PSCs [21–24]. It was reported that additives such as methylammonium chloride (MACl), MAH_2_PO_2_, and phenethylammonium thiocyanate (PEASCN) could regulate the crystallization of WBG perovskites and improve the film quality, thus mitigating the non-radiative recombination and reducing the *V*_OC_ deficits [25, 26]. Aside from suppressing the bulk defects, interface engineering by employing bulky alkylammonium halides or constructing 2D/3D heterojunctions was reported to effectively passivate the perovskite surface and reduce the *V*_OC_ deficits of WBG-PSCs [10, 27–34]. For instance, Zhou et al. treated the perovskite surfaces with benzylamine (BA), and the formed 2D BA_2_PbI_4_ (*n* = 1) passivated the surface defects of the 1.72-eV FA_0.15_Cs_0.85_Pb(I_0.73_Br_0.27_)_3_ perovskite (formamidinium is referred to as FA), enabling efficient WBG-PSCs with a high *V*_OC_ of 1.24 V [35]. Bu et al. employed phenylmethylamine bromide (PMABr) to form a hierarchically layered pure-2D (*n* = 1)/quasi-2D (*n* = 2) structure on top of the WBG perovskite and the additional Br^–^ further passivated the halide vacancies. These effects together reduced the *V*_OC_ deficit to 0.54 V and improved the stability of the devices [29]. Chen et al. reported that phenethylammonium iodide (PEAI) suppressed the accumulation of charge defects at the surface and grain boundaries of WBG perovskite by inhibiting the ion migration, leading to 19.07%-efficient PSC (with a bandgap of 1.73 eV) with reduced *V*_OC_ deficit of 0.48 V [27]. Other surface passivation molecules such as n-butylammonium bromide (BABr), guanidinium bromide (GABr), phenethylammonium chloride (PEACl), phenformin hydrochloride (PhenHCl), choline chloride, 1-butanethiol, etc. were demonstrated to improve the *V*_OC_ of WBG-PSCs to up to 1.31 eV and PCE up to 21.1% [9, 10, 30, 31, 34, 36, 37]. It is noted that these above-mentioned WBG-PSCs were fabricated using noble metal as the back electrodes.

Despite these exciting advances, there still lacks a deep understanding of the passivation mechanism of WBG perovskite. For instance, it was reported that aryl ammonium salt exhibited superior passivation ability than the corresponding thermal-annealing induced 2D perovskite for the normal-bandgap PSCs [3, 38, 39]. However, few reports have compared the passivation ability of 2D perovskites with the ionic salts on WBG perovskites. Moreover, the underlying mechanism of the hydrocarbonylammonium treatment was always attributed to the formation of 2D/3D heterojunction, a reduced defect density, and increased carrier lifetime. However, WBG perovskite and narrow-bandgap perovskite have different halogen components, thus the passivation mechanism may be different. Therefore, a deep understanding of how the bulky-cation structure of 2D perovskite affects the defect density of WBG perovskite is essential for designing more efficient surface passivation strategies to further boost the performance of WBG-PSCs and perovskite-based tandem solar cells. Additionally, most of the reported high-performance WBG-PSCs were fabricated using noble metal as the back electrode, but transparent electrodes are required for tandem solar cells. It is also worth mentioning that the transparent electrode has excellent reverse-bias stability [40].

Herein, the structure-performance relationship between the bulky-cation structure and the passivation ability on WBG perovskite was systematically studied by intentionally tailoring the skeleton of the bulky cations. Three alkylammonium chlorides, i.e., phenmethylammonium chloride (PMACl), phenethylammonium chloride (PEACl), and 1-naphthylmethylammonium chloride (NMACl), were employed to treat the WBG perovskite. Grazing-incidence wide-angle X-ray scattering and powder X-ray diffraction indicated that three kinds of passivating layers were obtained on top of the WBG perovskite. PMACl treatment led to the formation of pure-Cl 2D (PMA)_2_PbCl_4_. Post-treatment by PEACl, possessing one more methene unit than PMACl, formed a mixed I-Cl 2D (PEA)_2_PbI_*x*_Cl_4-*x*_ phase. NMACl treatment, with one additional aryl ring in the cation, led to NMAI salt through ion exchange. Carrier lifetime, defect density, and electroluminescence studies revealed that (PMA)_2_PbCl_4_ possessed the best passivation ability on the WBG perovskite due to a larger bandgap and higher conduction band minimum, which enabled excellent electron blocking at the top interface of the n-i-p WBG-PSCs. First-principle calculation and single-crystal X-ray diffraction results further provided a molecular mechanism of how bulky cation affected the structure of the 2D passivation layer. As a result, the semi-transparent WBG-PSC (indium tin oxide as the back electrodes) achieved a PCE of 18.60% with a high *V*_OC_ of 1.23 V.

## Experimental

### Materials

Tin (IV) oxide (SnO_2_, 15% in H_2_O colloidal dispersion) was purchased from Alfa Aesar. Lead iodide (PbI_2_, > 98%) and 1,4-butyrolactone (GBL, 99%) were bought from TCI. Lead bromide (PbBr_2_, > 99.99%), lead chloride (PbCl_2_, > 99.99%), methylammonium chloride (MACl, ≥ 99.5%), phenmethylammonium chloride (PMACl, ≥ 99.5%), and phenethylammonium chloride (PEACl, ≥ 99.5%) were received from Xi’an Polymer Light Technology Corporation. Cesium iodide (CsI, 99.9%), bis(trifluoromethanesulfonyl)imide (Li-TFSI, 99.95%), N,N-dimethylformamide (DMF, 99.8%), dimethyl sulfoxide (DMSO, 99.7%), isopropanol (IPA, 99.5%), chlorobenzene (CB, 99.8%), 4-tert-butylpyridine (4-TBP, 98%) and acetonitrile (ACN, 99.8%) were purchased from Sigma-Aldrich. Formamidinium iodide (FAI, 99.99%) and methylammonium iodide (MAI, 98.0%) were purchased from Greatcell Solar. 2,2’,7,7’-tetrakis[N,N-di(4-methoxyphenyl)amino]-9,9’-spirobifluorene (Spiro-OMeTAD, 99%) was bought from Shenzhen Feiming Technology Corporation. 1-naphthylmethylamine (NMA, 98%) was purchased from Innochem. Hydrochloric acid (HCl, 36.0% ~ 38.0%) was purchased from Sinopharm Chemical Reagent Co., Ltd. Molybdenum (VI) oxide (MoO_*x*_, > 99.998%) was purchased from Luminescence Technology Corp. ITO target (90 wt% In_2_O_3_, 10 wt% SnO_2_) was purchased from ZhongNuo Advanced Material (Beijing) Technology Co., LTD. All materials were used as received without further purification.

### Synthesis of NMACl

NMACl was synthesized according to the procedure reported in the literature [41]. Firstly, 16 mmol of NMA was mixed with 50 mL of tetrahydrofuran (THF) under the condition of the ice bath. Subsequently, 19.6 mmol of HCl was slowly added to the mixture. After that, the reaction solution was stirred at room temperature for 2 h. After all of the volatiles were removed by rotary evaporation, the crude products were washed with 20 mL of a mixed solvent of cold THF and dichloromethane (v/v = 1/3). Finally, the white powder of NMACl was obtained by recrystallization with ethanol.

### Fabrication of Perovskite Solar Cell

The ITO glass substrates were sequentially ultrasonically cleaned with detergent, deionized water, acetone, isopropanol, and ethanol for 20 min, respectively. After that, the substrates were dried with high-pressure nitrogen and immediately treated with plasma for 5 min to improve surface wettability. The SnO_2_ (5 wt% in H_2_O) precursor solution was spin-coated on the substrate at 4,000 rpm for 20 s and annealed at 150 °C in the air for 15 min. After cooling to room temperature, the substrate was transferred to the glove box for subsequent deposition steps.

The perovskite layer was prepared by the two-step sequential deposition method. First, the lead source precursor solution (PbI_2_: PbBr_2_: CsI = 0.9 M: 0.9 M: 0.09 M in 1 mL mixed solvent of DMF and DMSO (v/v = 9/1)) was spin-coated (Lebo science, EZ6) on the SnO_2_ substrate at 2,000 rpm for 30 s, and then annealed at 70 °C for 1 min. After cooling to room temperature, the organic salt solution (FAI: MACl: MAI = 0.37 M: 0.4 M: 0.15 M in 1 mL of IPA) was spin-coated onto the lead source layer at 0 rpm for 20 s and 1,700 rpm for 30 s and then annealed in the air at 150 °C for 15 min to form the perovskite layer.

For the post-treatment of the perovskite layer, PMACl, PEACl, or NMACl was dissolved in IPA and was dynamically spin-coated on the surface of the perovskite layer at 4,000 rpm for 30 s and then annealed at 100 °C for 5 min. The optimized solution concentrations for PMACl, PEACl, and NMACl were 3, 2, and 1 mg mL^−1^, respectively.

The Spiro-OMeTAD solution was prepared by mixing 90 mg of Spiro-OMeTAD, 36.1 μL of 4-TBP, and 21.8 μL of Li-TFSI (520 mg mL^−1^ in ACN) in 1 mL of CB. The solution was spin-coated at 3,000 rpm for 30 s without further annealing.

A ~ 20 nm MoO_*x*_ deposited by thermal evaporation was used as a buffer layer on the surface of the Spiro-OMeTAD layer. The transparent ITO electrode was prepared by magnetron sputtering with a deposition rate of 1.0 Å s^−1^ under 70 °C. The RF power was 50 W. Finally, the grid Ag electrode and MgF_2_ antireflection layer were evaporated on the surface of ITO and the back of glass substrates, respectively.

### Growth of 2D-Perovskite Single Crystals

(PMA)_2_PbCl_4_: First, 2 mmol of PMACl and 1 mmol of PbCl_2_ were dissolved in 1 mL of DMSO. Then, the solution was placed in acetone vapor at room temperature. After 2 days, white single crystals were obtained.

(PEA)_2_PbI_*x*_Cl_4-*x*_: 2 mmol of PEACl and 1 mmol of PbI_2_ were dissolved in 1 mL mixed solvent of GBL and DMSO (v/v = 1/1). The next steps were the same as the preparation method of (PMA)_2_PbCl_4_. Finally, yellowish single crystals were obtained.

### Characterizations

Grazing incidence wide-angle X-ray scattering (GIWAXS) measurements were conducted at a Xeuss SAXS/WAXS system (Xenocs, France) with a Pilatus3R 300 K detector. Scanning electron microscopy (SEM) images were obtained from a field-emission scanning electron microscope (JEOL JSM-7610F). X-ray diffraction (XRD) patterns were conducted on a SmartLab X-ray diffractometer (Rigaku Corporation) using Cu *K*_α_ (*λ* = 1.54 Å) as the radiation source. Atomic force microscopy (AFM) images were characterized by a Multimode 8 SPM system (Bruker). Ultraviolet–visible (UV–vis) absorption and steady-state photoluminescence (PL) spectra of perovskite films were performed on an instrument supplied by Xipu optoelectronics equipped with an integration sphere in the N_2_-filled glovebox. Time-resolved photoluminescence (TRPL) decay curves were measured by the FLS920 (Edinburgh Instruments Ltd). Ultraviolet photoelectron spectroscopy (UPS) spectra were carried out on a Thermo Fisher Scientific ESCALAB Xi + using a He-I_α_ UV light source. X-ray photoelectron spectroscopy (XPS) characterization was tested by Thermo Fisher Scientific K-Alpha + . Space-charge-limited-current (SCLC) curves were collected by a Keithley 2400 instrument in the dark. The *J-V* characteristics were tested through Keithley 2400 Source Meter in the glove box with a scan rate of 200 mV s^−1^ and a voltage step of 0.02 V under AM 1.5G illumination (Enlitech, AAA solar simulator). The light intensity was calibrated with a standard Si photodiode detector (equipped with a KG-5 filter). The active area of the device was 0.2 cm^2^ and a black metal mask with an effective of 0.12 cm^2^ was placed in front of the device. For the operating stability test, a white LED lamp was used as the light source and the light intensity was calibrated by a Si photodiode to 100 mW cm^−2^. The performance of the devices was tracked on a solar cell stability test system of PVLT-G8001M-256H (Suzhou D & Rinstruments Co., Ltd.). The external quantum efficiency (EQE) was measured by the Enlitech EQE measurement system (QER666). The Mott-Schottky analysis and transient photovoltage (TPV)were measured by the Zahner electrochemical workstation equipped with a transient electrochemical measurement unit (Fast CIMPS). Electrochemical impedance spectroscopy (EIS) was performed on the CHI660E electrochemical workstation. The device electroluminescence (EL) profiles and EQE were measured using a Keithley 2400 source meter and an EL measurement kit (Xipu optoelectronics).

### DFT Calculations

The density functional theory (DFT) calculations were performed using the Gaussian09 program package. The geometry optimizations were carried out at the B3LYP level with the 6-31G(d,p) basis set in the gas phase.

## Results and Discussion

### Regulating the Structure of the Passivation Layer on the Wide-bandgap Perovskite

**Fig. 1:**
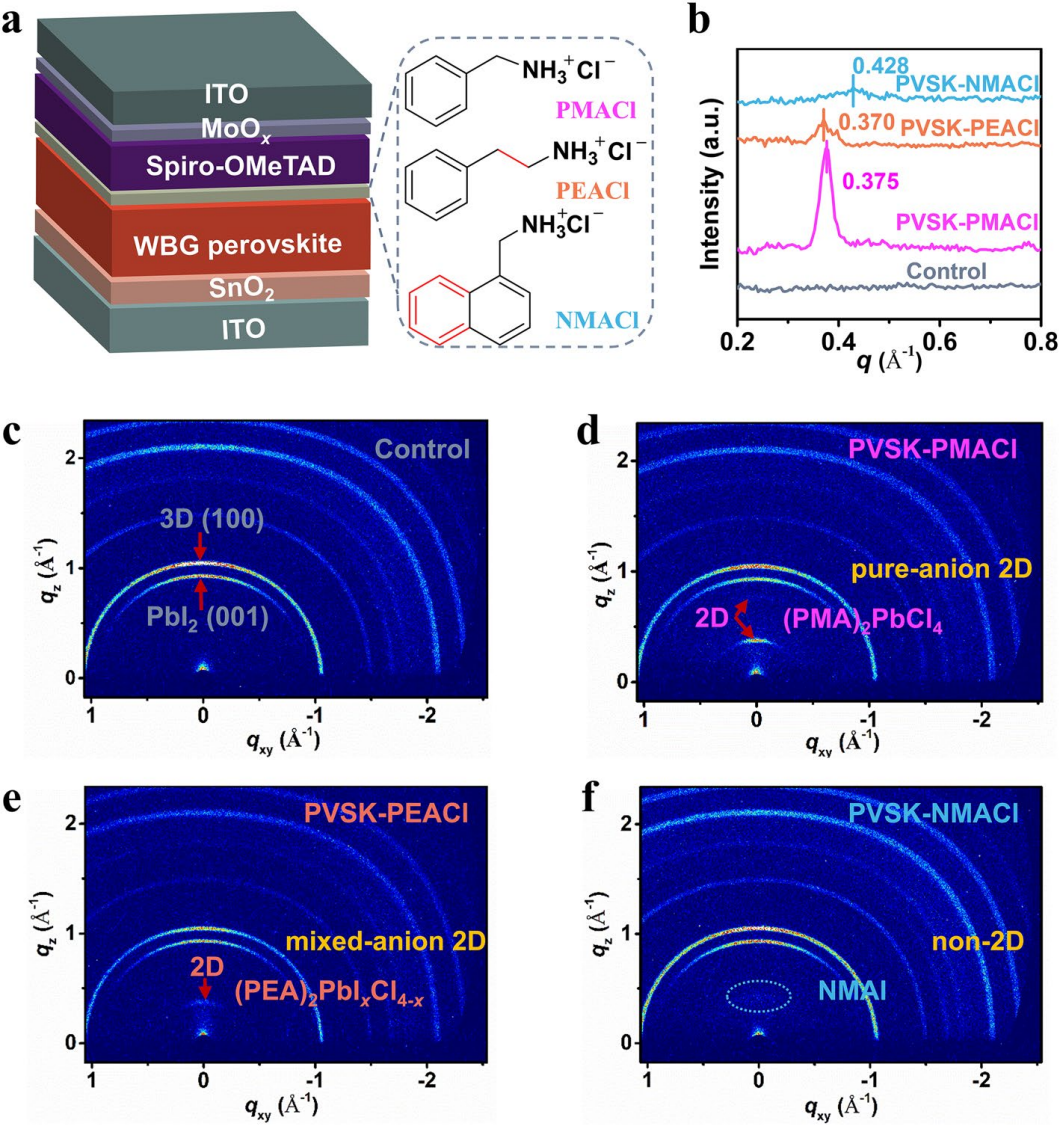
Influence of the bulky-cation structure on the composition of the passivation layer. **a** Schematic of the device structure of WBG-PSCs and the chemical structure of PMACl, PEACl, and NMACl. **b** Integrated GIWAXS profiles and **c-f** two-dimensional GIWAXS data of the control, PVSK-PMACl, PVSK-PEACl, and PVSK-NMACl films

The device structure of the WBG-PSCs adopted in this work consists of ITO/SnO_2_/WBG-perovskite/passivation layer/Spiro-OMeTAD/MoO_*x*_/ITO (Fig. 1a), where indium tin oxide is referred to as ITO and 2,2’,7,7’-tetrakis[N,N-di(4-methoxyphenyl)amino]-9,9’-spirobifluorene is referred to as Spiro-OMeTAD. Sputtered ITO was adopted as the transparent back electrode for semi-transparent PSCs. The composition of the WBG perovskite can be deduced to be Cs_0.05_FA_0.68_MA_0.27_Pb(I_0.57_Br_0.43_)_3_. Figure 1a also shows the molecular structure of PMACl, PEACl, and NMACl. Starting from PMACl, adding a methylene unit in the side chain of the cation gives PEACl, and replacing the benzene ring with the naphthalene ring gives NMACl. These molecules were spin-coated onto the WBG perovskite, followed by thermal annealing. Primary solar cell studies indicated that the optimal concentrations of PMACl, PEACl, and NMACl are 3, 2, and 1 mg mL^–1^, respectively (Figs. S1–S3). Unless otherwise stated, these optimal concentrations are used in the following studies. The perovskite films without post-treatment, treated with PMACl, PEACl, and NMACl are denoted as control, PVSK-PMACl, PVSK-PEACl, and PVSK-NMACl, respectively hereafter. GIWAXS was employed to investigate the perovskite structure transformation after the treatment. The integrated GIWAXS profiles (Fig. 1b) demonstrate that new phases are formed, with scattering vector *q* values of 0.375, 0.370, and 0.428 Å^–1^ for PVSK-PMACl, PVSK-PEACl, and PVSK-NMACl, respectively. Two-dimensional GIWAXS data (Fig. 1c–f) show that all the samples exhibit reflections at *q*≈0.9 and 1.0 Å^–1^, which can be attributed to the (001) crystal plane of PbI_2_ and the (100) plane of the 3D cubic perovskite plane, respectively, indicating a PbI_2_-rich perovskite film. The PVSK-PMACl, PVSK-PEACl, and PVSK-NMACl samples show new diffraction rings oriented in the out-of-plane direction (along the *q*_z_ direction, where *q*_z_ is the out-of-plane scattering vector), which is consistent with the integrated GIWAXS profiles. Powder XRD results (Fig. S4) further confirm the newly formed phase of PVSK-PMACl and PVSK-PEACl at ~ 5°. However, the signal of the PVSK-NMACl film may be under the detection limit.

To determine the composition of the new phases, more detailed control measurements were carried out. As shown in Fig. S5a–b, the 2D film prepared using the precursor of PbI_2_: PMACl = 1: 2 (molar ratio), i.e., (PMA)_2_PbI_*x*_Cl_4-*x*_, exhibits two sets of equidistant peaks that can be assigned to (PMA)_2_PbCl_4_ and (PMA)_2_PbI_4_ respectively. The inserted photographs in Fig. S5a show that the (PMA)_2_PbI_*x*_Cl_4-*x*_ exhibits an intermediary color, further supporting that (PMA)_2_PbI_*x*_Cl_4-*x*_ is a mixture of (PMA)_2_PbCl_4_ and (PMA)_2_PbI_4_. The XRD peak of the new phase of PVSK-PMACl appears at 5.32°, which is consistent with the diffraction pattern of (PMA)_2_PbCl_4_. Therefore, the new phase of PVSK-PMACl can be assigned to the pure-Cl 2D (PMA)_2_PbCl_4_ perovskite. This result indicates that the pure-Cl 2D phase with *n* = 1 is formed by treating the PbI_2_-rich WBG perovskite with PMACl. To clarify why (PMA)_2_PbI_4_ was absent on the surface of PVSK-PMACl, we tested the XRD patterns of (PMA)_2_PbI_*x*_Cl_4-*x*_ under different annealing temperatures (Fig. S6). Table S1 shows the relative diffraction intensity of the (001) crystal plane of (PMA)_2_PbCl_4_/(PMA)_2_PbI_4_. It shows that (PMA)_2_PbI_4_ is the dominant phase under low temperatures (from 40 to 90 °C), but it gradually transits to (PMA)_2_PbCl_4_ under high temperatures (100 to 120 °C). This result indicates that (PMA)_2_PbCl_4_ is favored after the annealing process at 100 °C for 5 min. Interestingly, XRD results in Figs. S5c–d and S7 show that the PVSK-PEACl film exhibits a single mixed-anion 2D (PEA)_2_PbI_*x*_Cl_4-*x*_ phase and this phase is stable in the whole temperature range from 40 to 130 °C. These results indicate that a mixed-anion I-Cl 2D (PEA)_2_PbI_*x*_Cl_4-*x*_ phase is formed on the surface. As can be seen from the inserted photographs, the film color of (PEA)_2_PbI_*x*_Cl_4-*x*_ is bright yellow. In addition, Table S2 demonstrates that different from halide doping in 3D perovskites, the 2D diffraction peak angle of the pure-I phase of (PEA)_2_PbI_4_ (5.46°) is larger than that of the pure-Cl phase of (PMA)_2_PbCl_4_ (5.32°) or the mixed I-Cl phase of (PEA)_2_PbI_*x*_Cl_4-*x*_ (5.22°). This tendency may be due to the difference in the interaction of cations by specific hydrogen bonds (N–H···X) and the disorder of the lead halogen plane, which affects the layer stacking [42]. For the PVSK-NMACl film, XRD patterns (Fig. S5e-f) show a diffraction peak at 6.10°, which can be assigned to the non-2D NMAI salt phase, indicating ion exchange occurred during the post-treatment. Note that 2D perovskite is not formed because of the higher formation energy of NMA-based 2D perovskite [38].

The above GIWAXS and powder XRD results support that by tailoring the molecular structure of the bulky cation while keeping the halide unchanged (Cl^–^), the composition of the passivation layer on the WBG perovskite can be regulated. PMACl treatment transformed to pure-anion 2D passivation, PEACl treatment led to mixed-anion 2D passivation, and NMACl treatment led to non-2D passivation.

SEM images show that the surface morphology of perovskite treated with different organic ammonium salts changes significantly (Fig. S8). For the PVSK-PMACl sample, many regular small particles are observed on the surface and at the grain boundaries of WBG perovskite, which is most likely the formed 2D (PMA)_2_PbCl_4_. The surface of the PVSK-PEACl film is covered by large-size 2D (PEA)_2_PbI_*x*_Cl_4-*x*_ plates. However, the PVSK-NMACl film shows a much-disordered surface morphology. The surface roughness of different perovskite films was examined by atomic force microscopy (AFM). The root-mean-square (RMS) roughness of the control, PVSK-PMACl, PVSK-PEACl, and PVSK-NMACl is 46.9, 37.9, 36.9, and 49.2 nm, respectively (Fig. S9). The decrease of roughness may be caused by the formation of 2D perovskites on the surface. The UV–vis absorption spectra of all perovskite films are almost the same (Fig. S10a), indicating that the passivating molecules do not change the perovskite lattice. The bandgap (*E*_g_) of WBG perovskite determined by the Tauc plot is about 1.73 eV (Fig. S10b).

### Characterizations of the Defect Passivation Effect

**Fig. 2:**
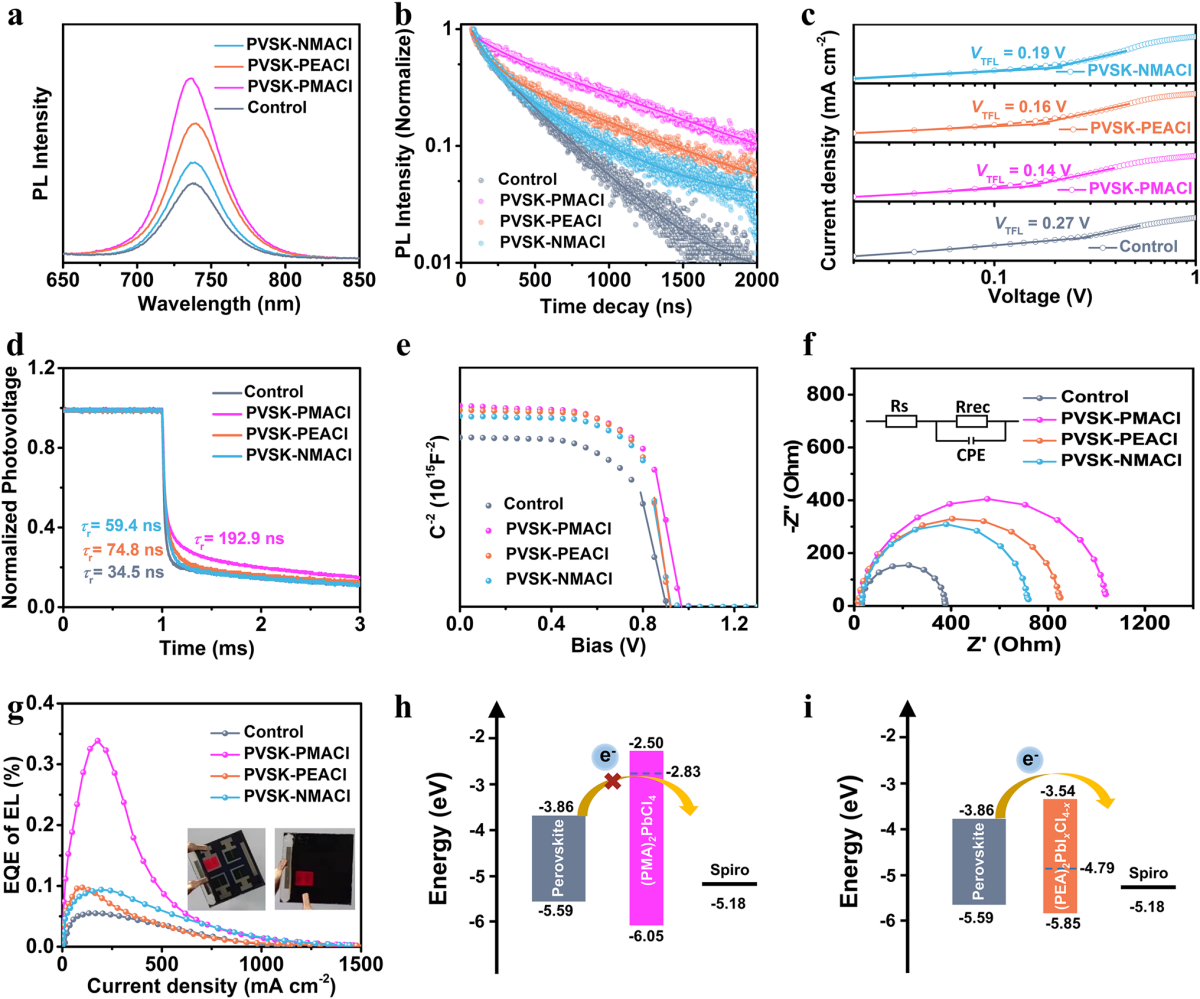
Characterization of defect passivation effect of PMACl, PEACl, and NMACl. **a** Steady-state photoluminescence spectra and **b** time-resolved photoluminescence decay curves of the control, PVSK-PMACl, PVSK-PEACl, and PVSK-NMACl films. **c** Space-charge-limited-current analysis of the electron-only devices measured under dark conditions. **d** Normalized transient photovoltage decay curves for the solar cells based on the control, PVSK-PMACl, PVSK-PEACl, and PVSK-NMACl films under light illumination with a power density of 100 mW cm^–2^. **e** Mott-Schottky analysis of PSCs in the dark using a frequency of 1 kHz. **f** Nyquist plots of the PSCs at a bias of 1.01 V under dark conditions. **g** EL-EQE of the devices while operating as LEDs. Insets: Photographs of the operating PSCs as LEDs. Schematic diagram of the energy-level alignment of WBG perovskite with** h** 2D (PMA)_2_PbCl_4_ and **i** 2D (PEA)_2_PbI_*x*_Cl_4-*x*_

To study the passivation effect of different organic ammonium salts on the WBG perovskite, a series of characterizations of perovskite films and PSCs were carried out. Steady-state photoluminescence (PL) measurements (Fig. 2a) excited from the passivator side using the sample structure of glass/perovskite/passivator were performed to investigate the carrier recombination within the perovskite films. Compared with the control film, the PL intensity of PVSK-PMACl, PVSK-PEACl, and PVSK-NMACl films are significantly enhanced, indicating suppressed non-radiative recombination. Additionally, the emission peak of the PVSK-PMACl film shows a distinct blue shift, which can be attributed to the passivation of shallow trap states on the perovskite surface by the amino functional groups of PMACl [31]. To verify this effect, TRPL decay measurements were used to study the dynamic characteristics of photogenerated carriers. The results are shown in Fig. 2b, and the detailed parameters fitted by the bi-exponential function are summarized in Table S3. The PVSK-PMACl film exhibits the longest average lifetime (*τ*_avg_) of 914.1 ns, while the *τ*_avg_ of control, PVSK-PEACl, and PVSK-NMACl films are 309.5, 675.9, and 369.4 ns, respectively. This result is consistent with the steady-state PL that PVSK-PMACl exhibits the highest PL intensity. In addition, PVSK-PMACl had the highest photoluminescence quantum efficiency (PLQY) value of 1.92% compared with other conditions (1.18% for Control, 1.69% for PVSK-PEACl, and 1.58% for PVSK-NMACl). These results imply that the pure-Cl 2D (PMA)_2_PbCl_4_ delivers the best defect passivation ability on the WBG perovskite. Space-charge-limited-current (SCLC) method was used to evaluate the trap density of perovskite films. Electron-only devices with the structure ITO/SnO_2_/Perovskite/[6, 6]-phenyl-C61-butyric acid methyl ester (PCBM)/Ag were prepared, and the dark *J-V* curves were measured (Fig. 2c). The trap-filled limit voltage (*V*_TFL_) is the voltage applied at the kink point, and the trap density can be calculated according to Eq. 1:

1 $$N_{{\text{t}}} = 2\varepsilon \varepsilon_{0} \frac{{V_{{{\text{TFL}}}} }}{{\left( {{\text{e}}L^{2} } \right)}}$$

where *N*_t_ is the trap density, ε is the relative permittivity, ε_0_ is the vacuum permittivity, e is the elementary charge, and *L* is the thickness of the perovskite film. According to the equation, the *V*_TFL_ is positively correlated with the trap density. As shown in Fig. 2c, the *V*_TFL_ of the control film is 0.27 V, while the *V*_TFL_ of PVSK-PMACl, PVSK-PEACl, and PVSK-NMACl samples is reduced to 0.14, 0.16, and 0.19 V, respectively. Therefore, the corresponding trap density decreased from 1.6 × 10^15^ cm^−3^ of the control film to the minimum of 8.5 × 10^14^ cm^−3^ of the PVSK-PMACl. The trap density of PVSK-PEACl and PVSK-NMACl film is also slightly suppressed, which is 9.7 × 10^14^ and 1.2 × 10^15^ cm^−3^, respectively.

To explore the effect of the passivating molecules on the charge transport and carrier recombination within the PSCs, more characterizations were carried out on the device level. From the cross-sectional SEM of the device (Fig. S11), we find that perovskites treated with organic ammonium salts exhibit vertical columnar grain arrangement, which will facilitate carrier transport in the vertical direction. TPV decay curves were used to detect the carrier recombination inside the device. As shown in Fig. 2d, the carrier recombination lifetime (*τ*_r_) is increased from 34.5 ms for the control to 192.9, 74.8, and 59.4 ms for the PVSK-PMACl, PVSK-PEACl, and PVSK-NMACl film, respectively. These results indicate that the passivating layer mitigates the non-radiative recombination rate at the perovskite/hole-transport-layer interface. We also measured the built-in potentials (*V*_bi_) of PSCs by Mott-Schottky analysis (Fig. 2e). *V*_bi_ of the devices based on the control, PVSK-PMACl, PVSK-PEACl, and PVSK-NMACl films are 0.90, 0.97, 0.92, and 0.92 V, respectively. Higher *V*_bi_ is conducive to more effective charge separation and reducing charge accumulation, which is beneficial to improving the photovoltage and reducing hysteresis. In addition, we further studied the carrier recombination behavior of the device by EIS (Fig. 2f). The PMACl passivated device shows the largest recombination resistance, indicating that the non-radiative recombination is effectively suppressed. The EQE of the PSCs working as light-emitting diodes (LEDs) can more directly reflect the non-radiative recombination, so we further measured the electroluminescence (EL) of the devices. The inserts of Fig. 2g are the photos of the lightened device. Due to the transparency of the back ITO electrode, both the top side and the bottom side can emit light. Figure 2g shows that the device based on PVSK-PMACl delivers the highest EL EQE, indicating the best effect of suppressing the non-radiative recombination. The corresponding EL spectra and *J-V* curves of the devices operating as LEDs are shown in Fig. S12. The above photophysics studies of the film and the carrier-transport studies of the devices support that the pure-Cl 2D (PMA)_2_PbCl_4_ possesses the best passivation effect on the WBG perovskite compared with the mixed I-Cl 2D (PEA)_2_PbI_*x*_Cl_4-*x*_ or the NMAI salt. In addition, XPS characterization was used to study the bonding environment on the surface of PVSK-PMACl film. From the C 1*s* spectra (Fig. S13a), it can be seen that the C=N characteristic peak of FA^+^ in PVSK-PMACl film shifts to lower binding energy, indicating that the benzene ring of PMACl provides an electron-rich environment for the 3D perovskite crystals. Moreover, in Fig. S13b, the characteristic peak of Pb 4*f* in the PVSK-PMACl film also shifts to lower binding energy than the control, indicating that Cl^–^ of PMACl could interact with undercoordinated Pb^2+^. In other words, PMACl can fill halide/organic cation vacancies and coordinate with undercoordinated Pb^2+^ of the 3D perovskite to efficiently passivate the defects.

To study how the 2D structure affects the defect passivation ability of the WBG perovskite, ultraviolet photoelectron spectroscopy (UPS) was used to investigate the band alignment between the WBG 3D perovskite and different 2D perovskites (PMA)_2_PbCl_4_ and (PEA)_2_PbI_*x*_Cl_4-*x*_. Combining the UPS spectra in Fig. S14 and the Tauc plots in Fig. S10b, the valence band maximum (VBM) and conduction band minimum (CBM) of 3D perovskites, (PMA)_2_PbCl_4_, (PEA)_2_PbI_*x*_Cl_4-*x*_ can be determined, and the energy level alignment is shown in Fig. 2h–i. The VBM of the WBG perovskite (− 5.59 eV) is higher than that of (PMA)_2_PbCl_4_ (− 6.05 eV) and (PEA)_2_PbI_*x*_Cl_4-*x*_ (− 5.86 eV), and the CBM of the WBG perovskite (− 3.86 eV) is lower than that of (PMA)_2_PbCl_4_ (− 2.50 eV) and (PEA)_2_PbI_*x*_Cl_4-*x*_ (− 3.54 eV), both form type-I band alignment with the WBG perovskite, which enables effective defect passivation ability [43]. (PMA)_2_PbCl_4_ has a larger bandgap of 3.55 eV than (PEA)_2_PbI_*x*_Cl_4-*x*_ (2.31 eV) and higher CBM, which can act as an electron-transport barrier and mitigate the electron–hole recombination at the perovskite/hole-transport-layer interface.

Based on the above analysis, PMACl, PEACl, and NMACl have defect passivation ability for PbI_2_-rich WBG perovskite films. Among them, the superior passivation ability of the PMACl treatment is most likely due to the united effects of the more ordered distribution of the 2D phase on the WBG perovskite surface and the improved interfacial energy-level arrangement. The performance of PVSK-PEACl is better than that of control and PVSK-NMACl, but inferior to that of PVSK-PMACl, the possible reason is that the formed 2D perovskite has passivation ability on the perovskite surface but also leads to undesirable aggregation. The performance of NMACl passivation is only better than that of control, mainly due to the passivation effect of the disordered NMAI salt formed on the perovskite surface being inferior. Therefore, the defect passivation ability is in the sequence of PVSK-PMACl > PVSK-PEACl > PVSK-NMACl > Control.

### Molecular Mechanism of the Formation of Pure-Cl 2D Phase

**Fig. 3:**
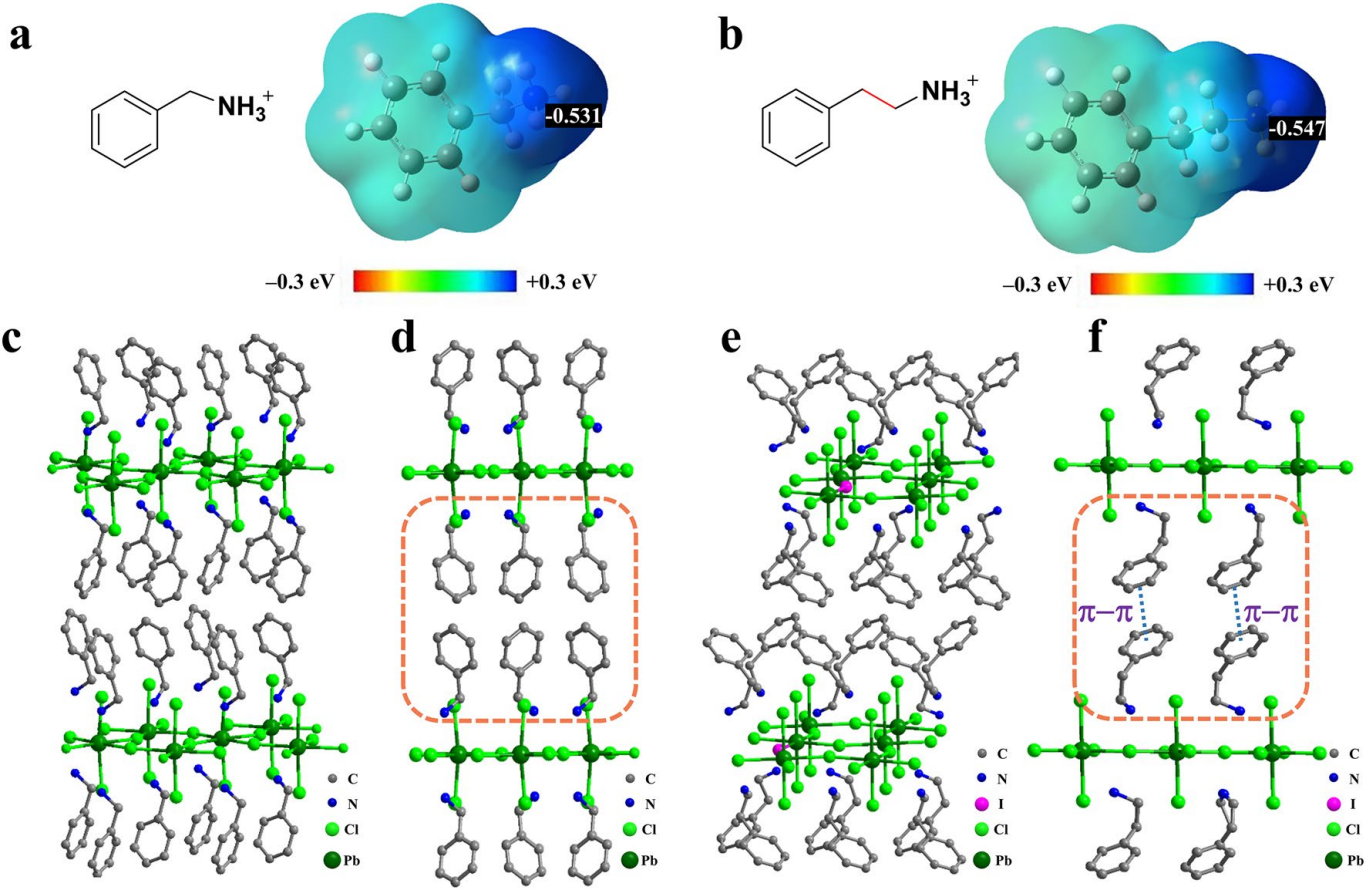
Charge distribution of **a** PMA^+^ and **b** PEA^+^ calculated by density functional theory (DFT). Crystal structure of **c**, **d** (PMA)_2_PbCl_4_ and **e**, **f** (PEA)_2_PbI_*x*_Cl_4-*x*_

To understand the molecular mechanism of how cation structure regulates the halide composition of the 2D passivating layer, first-principle calculations of the cations and single-crystal XRD measurements of the 2D perovskite were carried out. The calculated charge distribution reveals that the N atom in PEA^+^ possesses a more negative localized Mulliken charge (− 0.547) than PMA^+^ (− 0.531) (Fig. 3a-b), indicating larger electronegativity of the N atom in PEA^+^. This phenomenon is most likely due to the conjugation effect of the N atom in the benzyl group being stronger than that in the phenyl group. Therefore, PEA^+^ can more easily form hydrogen bonds with I^–^ than that of PMA^+^, leading to a stable mixed I-Cl 2D phase. However, PMACl treatment results in a pure-Cl 2D phase due to unstable hydrogen bonding of N–H‧‧‧I. We prepared PMA-based and PEA-based 2D perovskites single crystals and measured their crystal structure. The crystal structure and the detailed crystal parameters are shown in Fig. 3c–f and Table 1. The crystal structure confirms that PEACl treatment forms a mixed I-Cl 2D phase while PMACl treatment forms a pure-Cl 2D phase. Symmetry reduction is observed from a Cmc2_1_ space group of (PMA)_2_PbCl_4_ to a P-1 space group of (PEA)_2_PbI_*x*_Cl_4-*x*_. Moreover, the side view of the crystal structure (Fig. 3c–f) shows that two adjacent PEA^+^ layers can form *π*–*π* interaction due to the paralleled stacking, which may further contribute to stabilizing the I-Cl mixed 2D phase. However, this *π*-*π* interaction may lead to undesirable aggregation of the passivator on the perovskite surface [44].

**Table 1:** Crystal parameters of (PMA)_2_PbCl_4_ and (PEA)_2_PbI_*x*_Cl_4-*x*_

Perovskite	Space group	Crystal system	*a* (Å)	b (Å)	c (Å)	*α* (°)	*β*(°)	*γ*(°)
(PMA)_2_PbCl_4_	Cmc2_1_	Orthorhombic	33.157	7.775	7.652	90	90	90
(PEA)_2_PbI_*x*_Cl_4-*x*_	P-1(2)	Triclinic	5.510	5.582	16.858	80	85	90

### Performance of Semi-Transparent Wide-Bandgap Perovskite Solar Cells

**Fig. 4:**
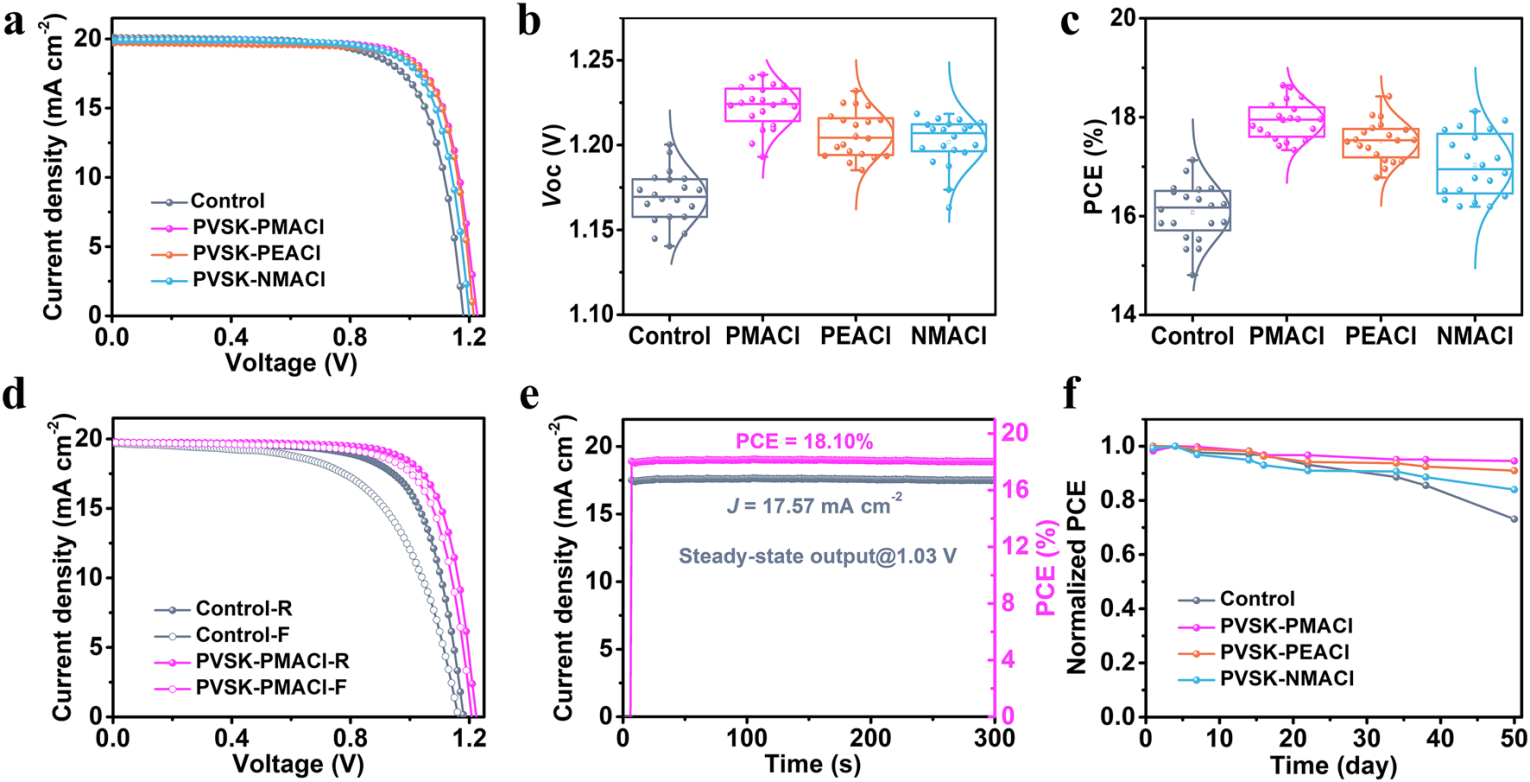
Photovoltaic performance of the WBG-PSCs. **a** Typical *J-V* curves of the WBG-PSCs based on the control, PVSK-PMACl, PVSK-PEACl, and PVSK-NMACl films under one-sun (100 mW cm^–2^) conditions. **b, c** Statistical results of *V*_OC_ and PCE. Each group contains 20 PSCs. **d** *J-V* curves of the devices based on the control and PVSK-PMACl films measured by forward and reverse scans. **e** Steady-state power output at the maximum power point of the best device at 1.03 V for 300 s. **f** Stability of the WBG-PSCs. The devices were stored in the air with a relative humidity of ~ 10% and a temperature of ~ 25 °C

The reduction of trap states and the favorable energy-level alignment are beneficial to suppressing the non-radiative recombination, thereby improving the photovoltaic performance of the WBG-PSCs. Figure 4a shows the *J-V* curves of the champion devices, and Table 2 summarizes the corresponding detailed photovoltaic parameters. Compared to the control with a PCE of 17.13% and a *V*_OC_ of 1.18 V, the PCE of PSCs based on PVSK-PMACl, PVSK-PEACl, and PVSK-NMACl films are increased to 18.60%, 18.42%, and 18.12%, respectively. The corresponding *V*_OC_ are increased to 1.23, 1.21, and 1.20 V, respectively. The performance of the device based on PVSK-PEACl is inferior to that of the device based on PVSK-PMACl. On the one hand, the large-size 2D perovskite formed by PEACl treatment is not well distributed in the grain boundaries; on the other hand, the mixed I-Cl 2D perovskite is insufficient in blocking the electrons, leading to partial carrier recombination. The device performance of PVSK-NMACl film is lower than that of PVSK-PMACl and PVSK-PEACl. One possible reason is probably that the disordered NMAI salt can’t deliver sufficient passivation. Incident photon-to-electron conversion efficiency (IPCE) spectra (Fig. S15) were measured, and the integrated short-circuit current density (*J*_SC_) of different devices was compared with the *J*_SC_ extracted from the *J-V* curves (Table 2). The minor discrepancy confirmed the reliability of the *J-V* test. The statistical distribution of *V*_OC_, PCE, *J*_SC_ and fill factor (FF) of 20 devices for each group show that the devices based on PVSK-PMACl possess the best performance, which reflects the superior passivation effect of the pure-Cl 2D phase (Figs. 4b-c and S16). The highest *V*_OC_ of the PMACl-passivated device reaches 1.24 V, indicating a *V*_OC_ deficit of 0.49 V for the 1.73-eV perovskite solar cells, which is one of the best performances among the semi-transparent WBG PSCs. The forward and reverse scanned *J-V* curves of the devices based on control, PVSK-PMACl, PVSK-PEACl, and PVSK-NMACl films are shown in Figs. 4d and S17. The hysteresis factor (HI) is calculated by Eq. 2:

2 $${\text{HI}}\left( {\text{\% }} \right) = \left( {{\text{PCE}}_{{{\text{reverse}}}} - {\text{PCE}}_{{{\text{forward}}}} } \right)/{\text{PCE}}_{{{\text{reverse}}}} \times 100{\text{\% }}$$

and the detailed parameters are listed in Table S4. The HI of the control is 15.52% (PCE_reverse_ of 16.52% and PCE_forward_ of 14.00%), which is significantly reduced to 4.19% (PCE_reverse_ of 18.38% and PCE_forward_ of 17.62%) after PMACl treatment, indicating that the ion migration is probably suppressed after passivation. The performance of the semi-transparent device with the light incident from the MoO_*x*_/ITO side and the performance of the opaque device with a silver electrode is shown in Fig. S18 and Table S5. The performance difference between light incidents from the MoO_*x*_/ITO side and the glass/ITO side is due to different *J*_SC_. From Fig. S18b, the reduction of *J*_SC_ on the MoO_*x*_/ITO side is mainly due to the absorption of short-wavelength light by the Spiro-OMeTAD. The semi-transparent cell with the light incident from the MoO_*x*_/ITO side and the opaque device obtained PCE of 16.42% and 19.62%, respectively. We note that in the 4-terminal (4T) tandem solar cells, the light can incident from the glass/ITO side of the semi-transparent cell, so the semi-transparent n-i-p device can be used to fabricate high-performance 4T tandems.

**Table 2:** Photovoltaic parameters of champion devices based on the control, PVSK-PMACl, PVSK-PEACl, and PVSK-NMACl films

Sample	*J*_SC_ (mA cm^−2^)	*V*_OC_ (V)	FF (%)	PCE (%)
Control	20.11	1.18	72.17	17.13
PVSK-PMACl	19.87	1.23	76.31	18.60
PVSK-PEACl	19.75	1.21	76.78	18.42
PVSK-NMACl	19.90	1.20	75.87	18.12

Stability is another parameter for evaluating the device performance. The short-term operational stability of devices is obtained at a fixed voltage near the maximum power point (MPP) under AM 1.5G solar simulator. As shown in Fig. 4e, the stabilized power output (SPO) of the optimal performance device is 18.10%, which is higher than that based on the control (16.33%), PVSK-PEACl (17.22%), and PVSK-NMACl (16.69%) (Fig. S19a–c), respectively. For long-term stability, we monitored the efficiency of the devices stored in the air (the relative humidity is ~ 10%, and the temperature is ~ 25 °C) for 50 days (Fig. 4f). The PCE of the control device drops to 73% of its initial PCE, while that of devices based on the PVSK-PMACl, PVSK-PEACl, and PVSK-NMACl films retain 94%, 90%, and 84% of their initial PCEs. To study the operational stability of the devices, the photovoltaic parameters of the devices operating at maximum power point (MPP) under 1 sun illumination were collected (Fig. S20). The results demonstrated that the operational stability of the devices was improved after passivation. The PVSK-PMACl device can maintain 80% of the initial efficiency after 350 h of MPP operation, while the control decays to 80% of the initial efficiency after 100 h. The much-improved stability of the PVSK-PMACl devices may originate from the reduced defect density and improved humidity resistance due to the formation of the pure-Cl 2D phase. In addition, the light transmission of the device was also tested. As shown in Fig. S21, the devices show good light transmission in the near-infrared region, which is comparable with the reported semi-transparent solar cells in high-performance perovskite/silicon tandem solar cells [45–47]. Therefore, our strategy is suitable for improving the performance of perovskite-based tandem solar cells.

## Conclusions

In summary, we demonstrate that the halogen composition of the 2D perovskite passivation layer can be regulated by tailoring the structure of bulky ammonium cations. Due to the weak intermolecular interaction and relatively low cation-halide hydrogen bonding strength, the PMA^+^ forms a pure Cl-2D phase on the surface of WBG perovskite. In contrast, the PEA^+^ forms I-Cl mixed 2D perovskite, and the NMA^+^ forms non-2D perovskite (salt). The 2D (PMA)_2_PbCl_4_ perovskite has a large bandgap and a high conduction-band-minimum, which can form improved type-I energy level alignment with the 1.73-eV WBG perovskites. Therefore, the *V*_OC_ deficits of semi-transparent WBG-PSCs (indium tin oxide as the back electrodes) can be reduced to 0.49 V, which is comparable with the reported state-of-the-art *V*_OC_ deficits in WBG-PSCs with metal electrodes. Due to these effects, 18.60%-efficient semi-transparent WBG-PSCs are obtained. We believe this study will provide valuable insights into the designing principle of effective passivation strategies for WBG perovskites and perovskite-based tandem solar cells.

### Supplementary Information

Below is the link to the electronic supplementary material.

Supplementary file1 (PDF 2083 KB)
