# Supplementary material for: Efficient Semi-Transparent Wide-Bandgap Perovskite Solar Cells Enabled by Pure-Chloride 2D-Perovskite Passivation
Source: Nanomicro Lett. 2023 Apr 30;15:111. doi: 10.1007/s40820-023-01090-w (PMC10149431; doi:10.1007/s40820-023-01090-w)
Supplement: Supplementary file 1 — Supplementary file1 (PDF 2083 KB) [file 40820_2023_1090_MOESM1_ESM.pdf]

Supporting Information for

Efficient Semi-Transparent Wide-Bandgap Perovskite Solar Cells Enabled by Pure-Chloride 2D-Perovskite Passivation

Liu Yang[1, #], Yongbin Jin[1, #], Zheng Fang[2, #], Jinyan Zhang[3], Ziang Nan[4], Lingfang Zheng[1], Huihu Zhuang[3], Qinghua Zeng[3], Kaikai Liu[1], Bingru Deng[1], Huiping Feng[1], Yujie Luo[1], Chengbo Tian[1], Changcai Cui[2], Liqiang Xie[1, *], Xipeng Xu[2, *], and Zhanhua Wei[1, *]

[1]Xiamen Key Laboratory of Optoelectronic Materials and Advanced Manufacturing, Institute of Luminescent Materials and Information Displays, College of Materials Science and Engineering, Huaqiao University, Xiamen 361021, P. R. China

[2]MOE Engineering Research Center for Brittle Materials Machining, Institute of Manufacturing Engineering, College of Mechanical Engineering and Automation, Huaqiao University, Xiamen 361021, P. R. China

[3]Gold Stone (Fujian) Energy Company Limited, Quanzhou 362005, P. R. China

[4]Collaborative Innovation Center of Chemistry for Energy Materials, Department of Chemistry, College of Chemistry and Chemical Engineering, Xiamen University, Xiamen 361005, P. R. China

# Liu Yang, Yongbin Jin and Zheng Fang contributed equally to this work.

*Corresponding authors. E-mail: lqxie@hqu.edu.cn (Liqiang Xie); xpxu@hqu.edu.cn (Xipeng Xu); weizhanhua@hqu.edu.cn (Zhanhua Wei)

Supplementary Figures and Tables

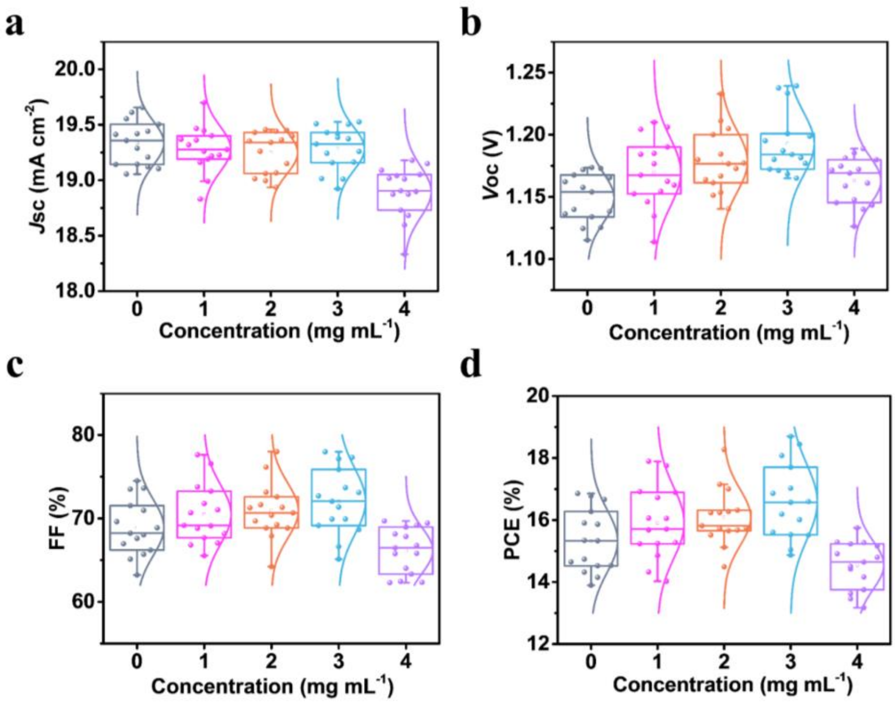

Fig. S1 Statistical distribution of photovoltaic metrics of PSCs passivated with different PMACl concentrations. **a** J_{SC}, **b** V_{OC}, **c** FF, and **d** PCE

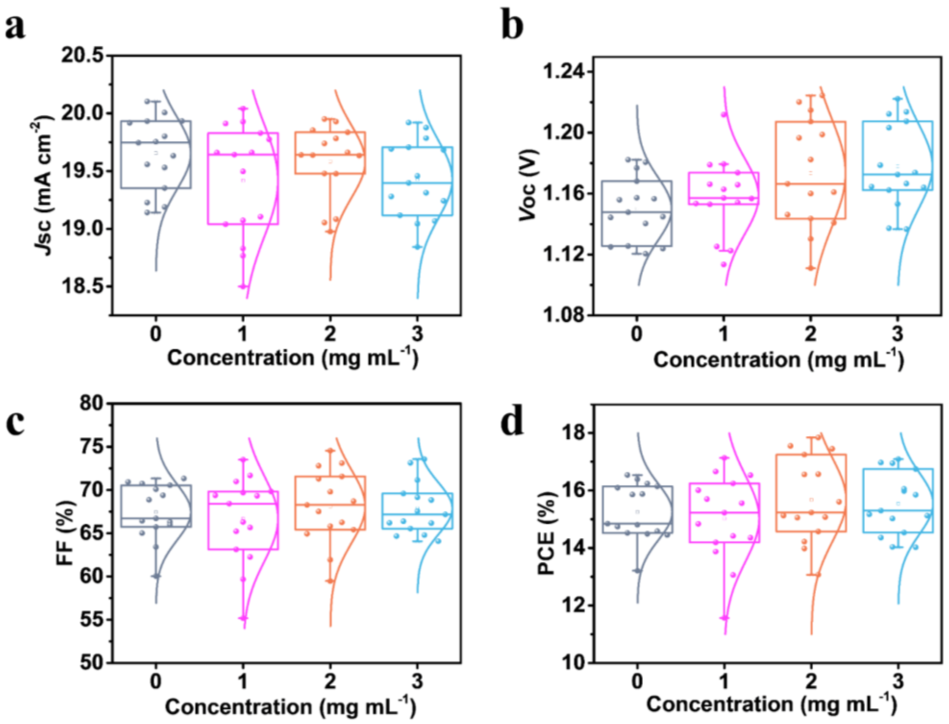

Fig. S2 Statistical distribution of photovoltaic metrics of PSCs passivated with different PEACl concentration. **a** J_{SC}, **b** V_{OC}, **c** FF, and **d** PCE

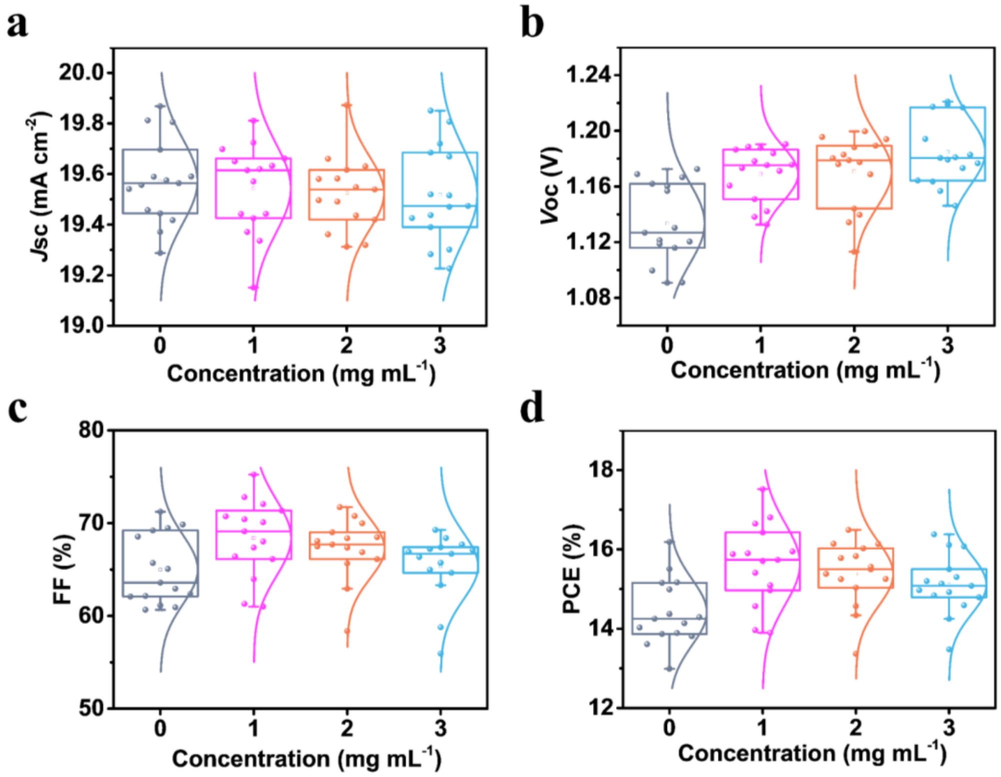

Fig. S3 Statistical distribution of photovoltaic metrics of PSCs passivated with different NMACl concentration. **a** J_{SC}, **b** V_{OC}, **c** FF, and **d** PCE

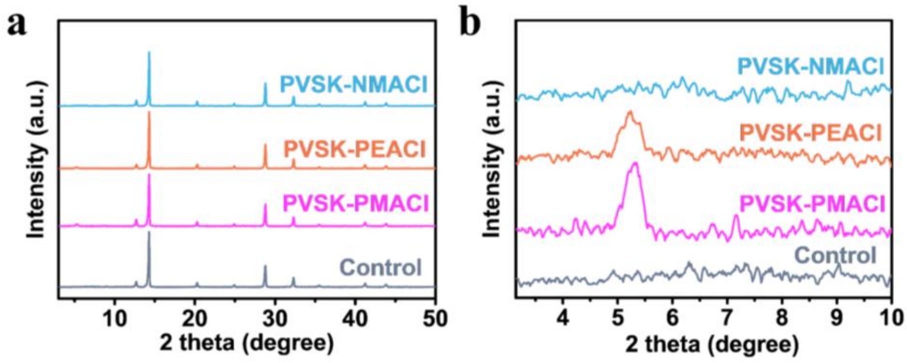

Fig. S4 a XRD patterns of the control, PVSK-PMACl (3 mg mL^{-1}), PVSK-PEACl (2 mg mL^{-1}), and PVSK-NMACl (1 mg mL^{-1}) films. **b** Enlarged XRD patterns of **a** in the low diffraction angle region

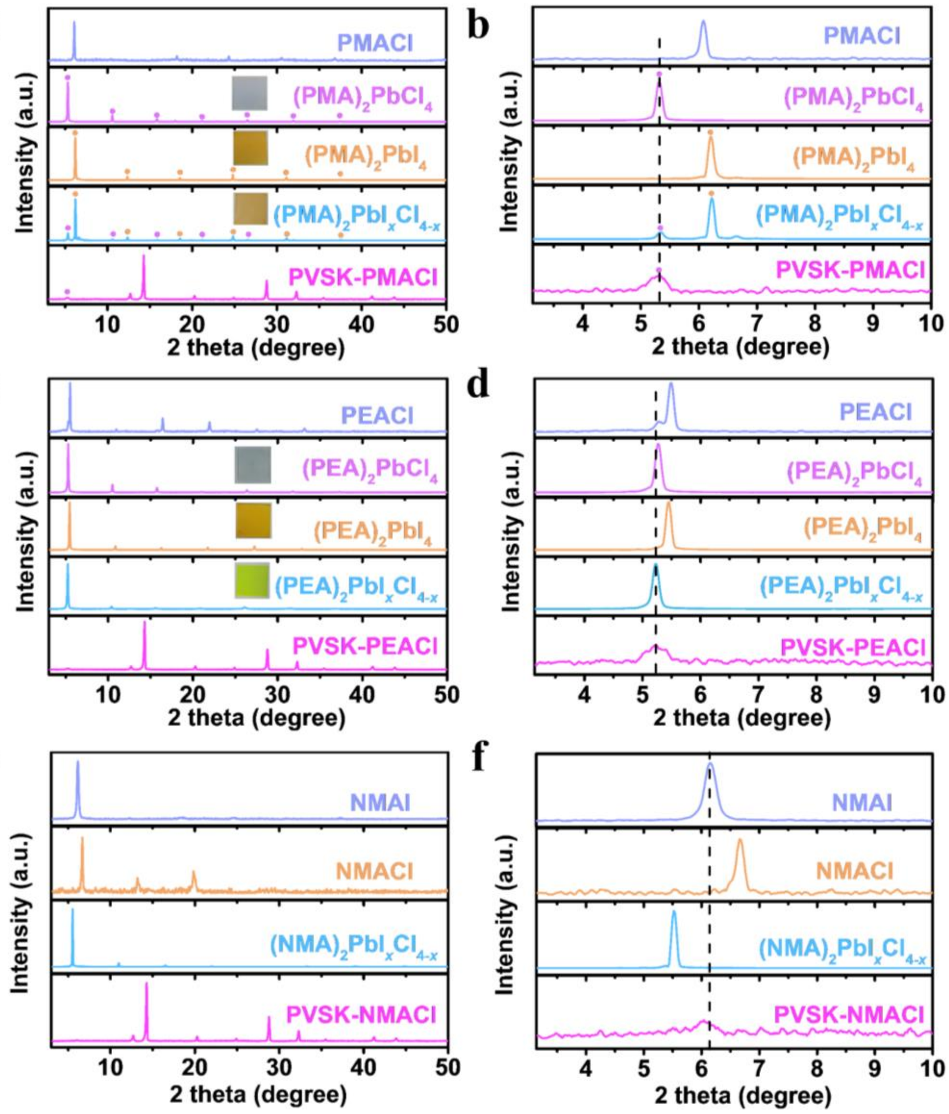

Fig. S5 a, b XRD patterns of PVSK-PMACl (3 mg mL^{-1}), $(PMA)_2PbI_xCl_{4-x}$, PMA_2PbI_4, $(PMA)_2PbCl_4$, and PMACl. Insets: Photographs of the corresponding films. **c, d** XRD patterns of PVSK-PEACl (2 mg mL^{-1}), $(PEA)_2PbI_xCl_{4-x}$, $(PEA)_2PbI_4$, $(PEA)_2PbCl_4$, and PEACl. Insets: Photographs of the corresponding films. **e, f** XRD patterns of PVSK-NMACl (3 mg mL^{-1}), $(NMA)_2PbI_xCl_{4-x}$, NMACl, and NMAI

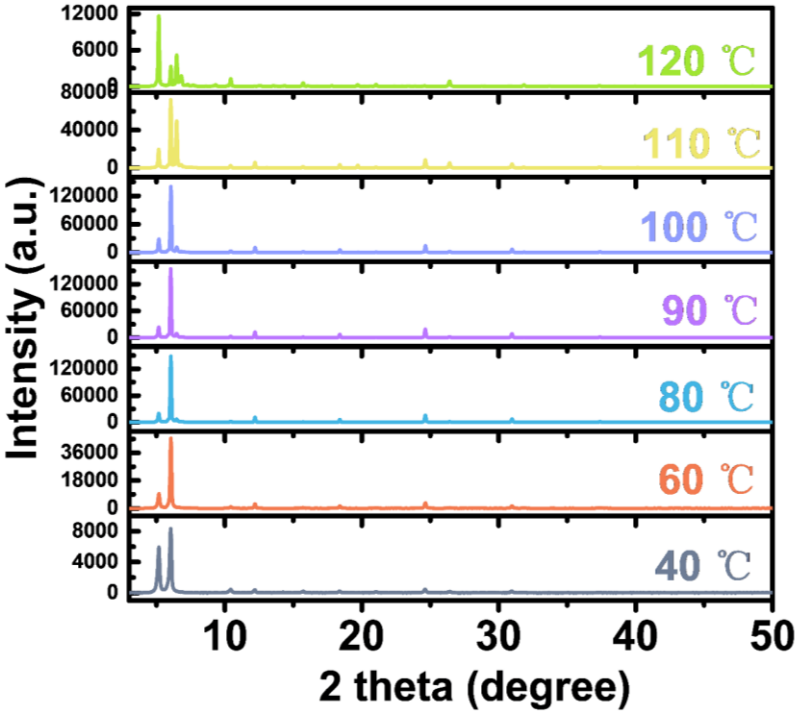

Fig. S6 XRD patterns of 2D $(PMA)_2PbI_xCl_{4-x}$ at different annealing temperatures

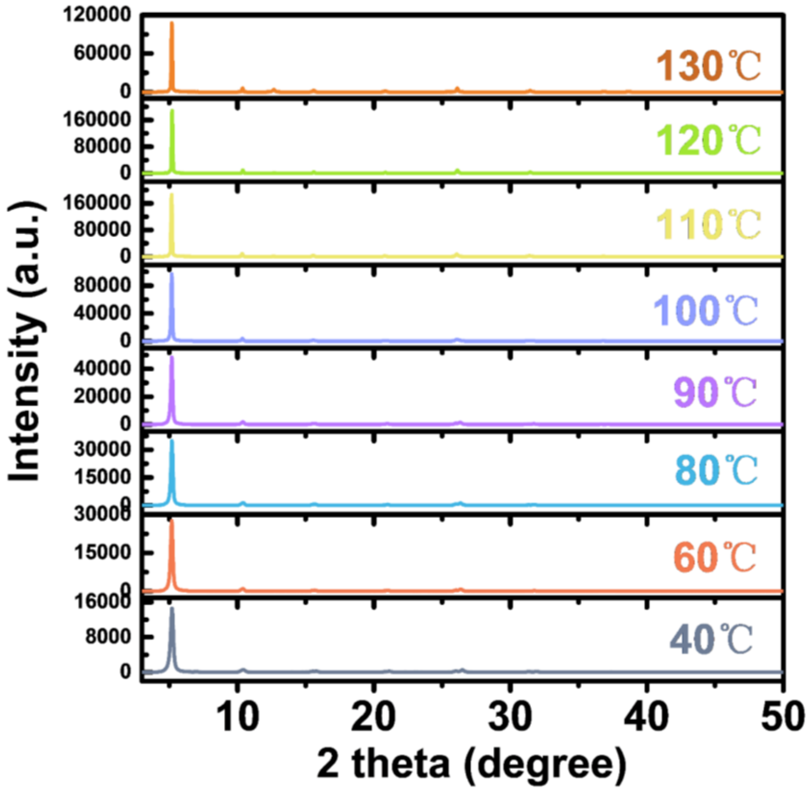

Fig. S7 XRD patterns of 2D $(PEA)_2PbI_xCl_{4-x}$ at different annealing temperatures

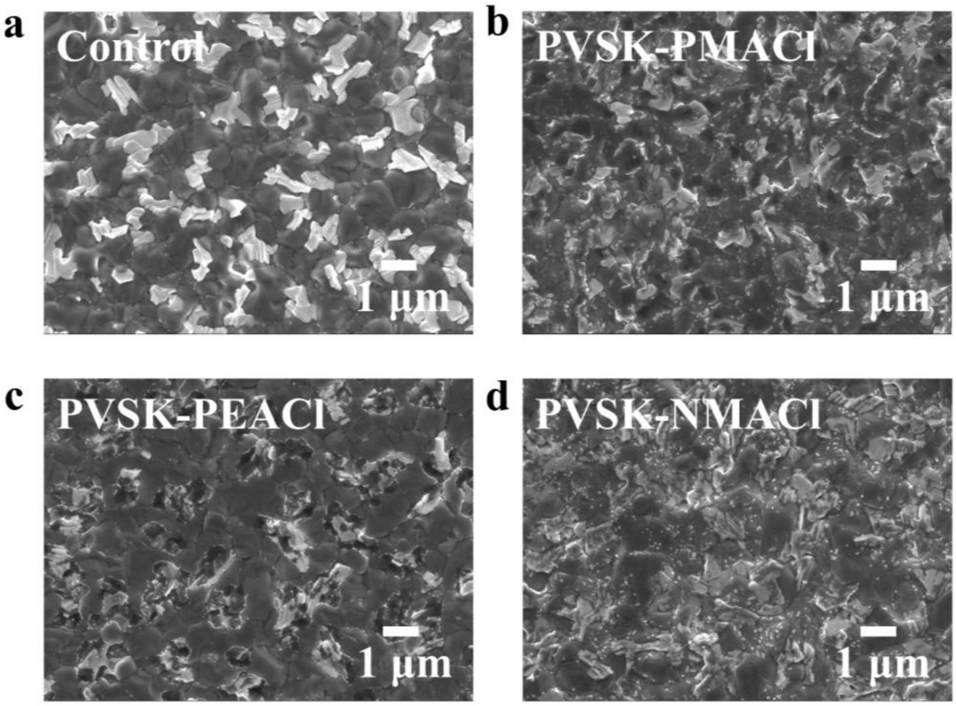

Fig. S8 Scanning electron microscopy (SEM) images of the **a** control, **b** PVSK-PMACl, **c** PVSK-PEACl, and **d** PVSK-NMACl films

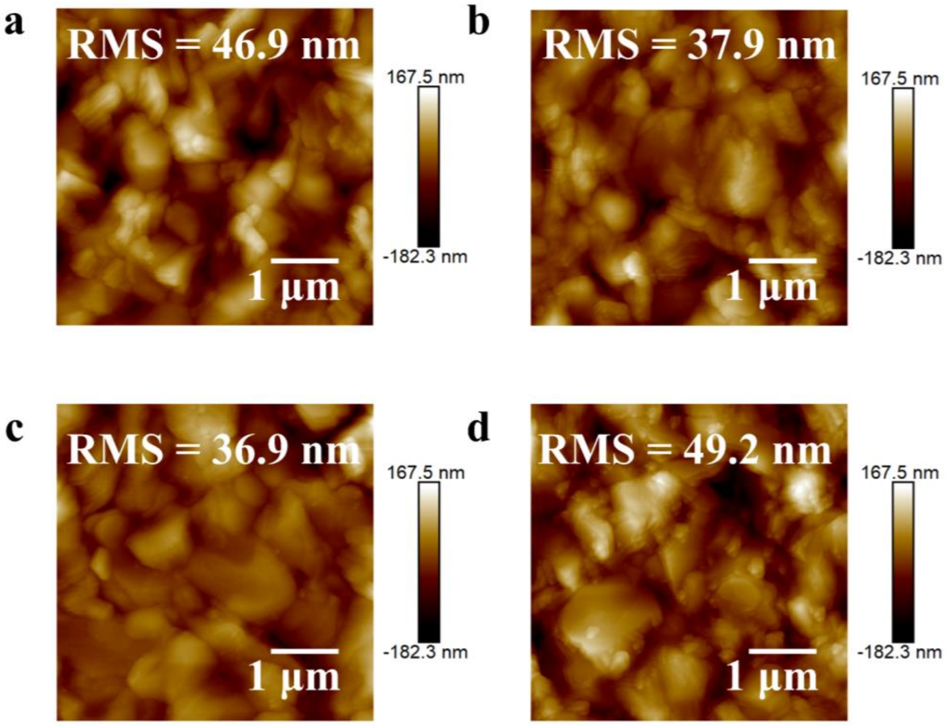

Fig. S9 Atomic force microscopy (AFM) images of the **a** control, **b** PVSK-PMACl, **c** PVSK-PEACl, and **d** PVSK-NMACl films

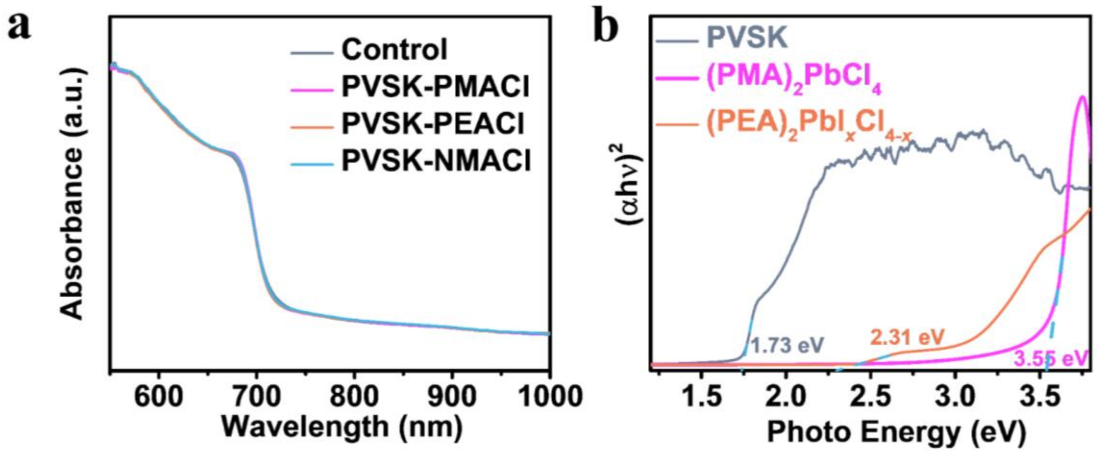

Fig. S10 **a** UV-vis absorption spectra of the control, PVSK-PMACl, PVSK-PEACl, and PVSK-NMACl films. **b** Tauc plot of the control, 2D $(PMA)_2PbCl_4$, and 2D $(PEA)_2PbI_xCl_{4-x}$ films

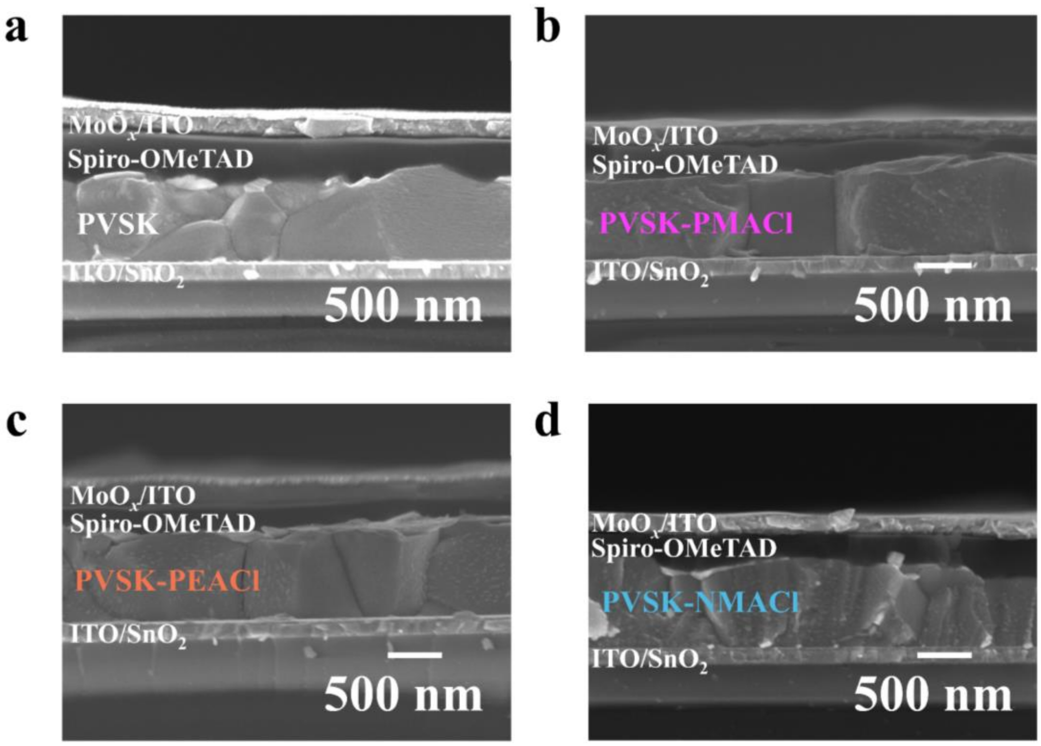

Fig. S11 Cross-sectional SEM images of the device based on the **a** control, **b** PVSK-PMACl, **c** PVSK-PEACl, and **d** PVSK-NMACl films

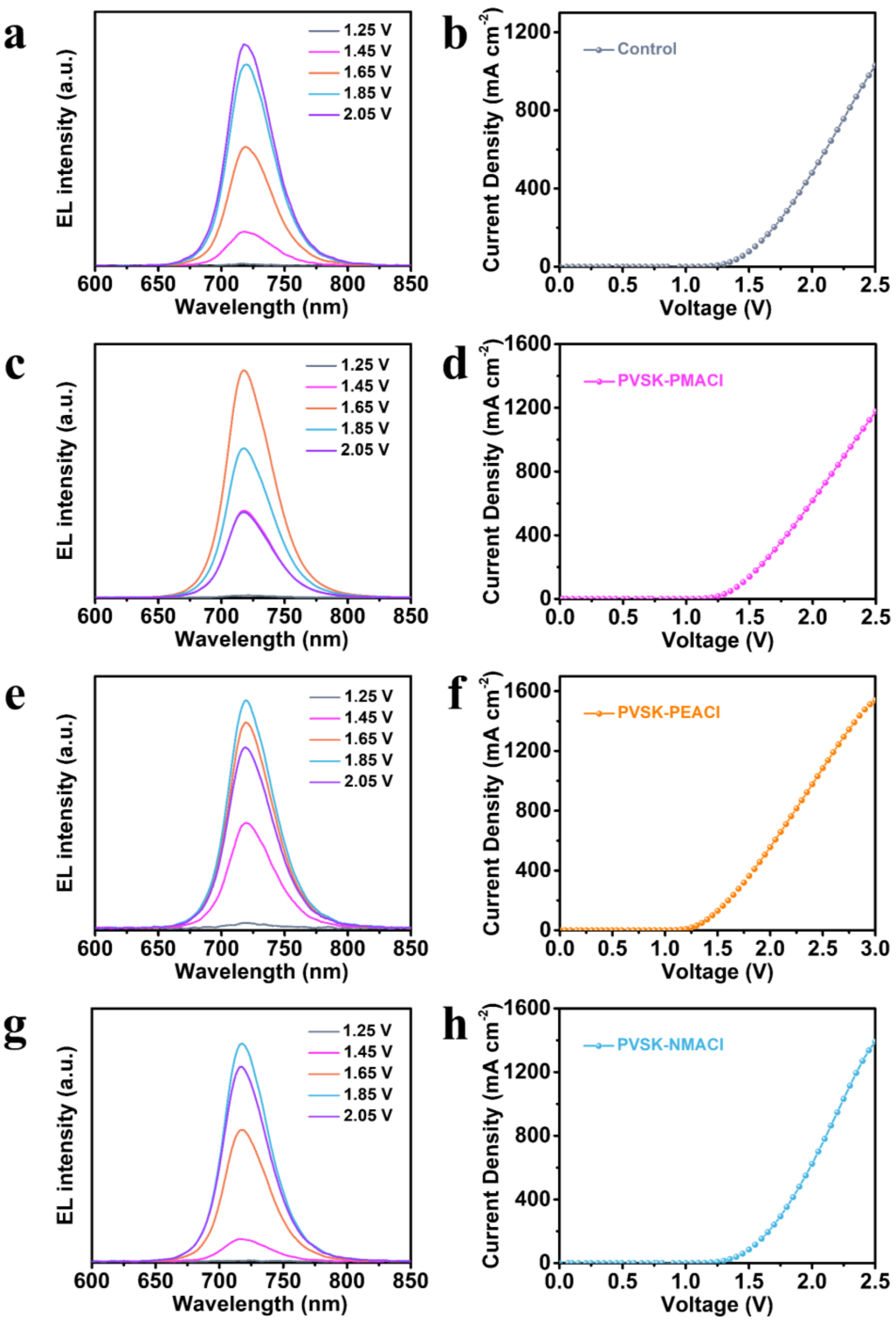

Fig. S12 a, c, e, and **g** EL spectra of the devices based on the control, PVSK-PMACl, PVSK-PEACl, and PVSK-NMACl films operating as LEDs. **b, d, f,** and **h** *J-V* curves of the devices based on the control, PVSK-PMACl, PVSK-PEACl, and PVSK-NMACl films

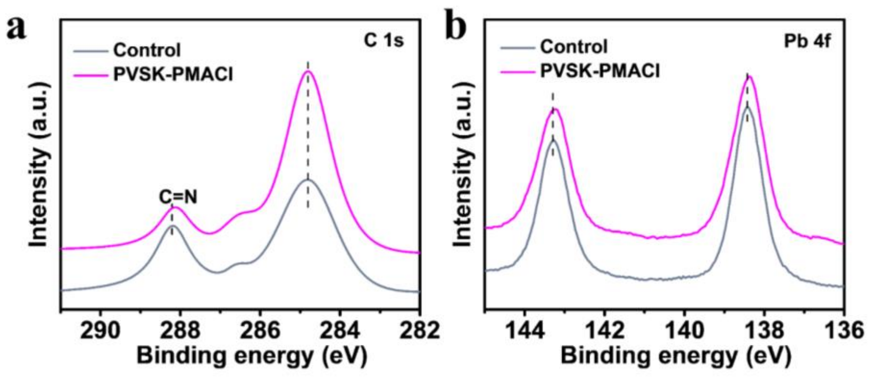

Fig. S13 a C 1s and **b** Pb 4f XPS spectra of the control and PVSK-PMACl

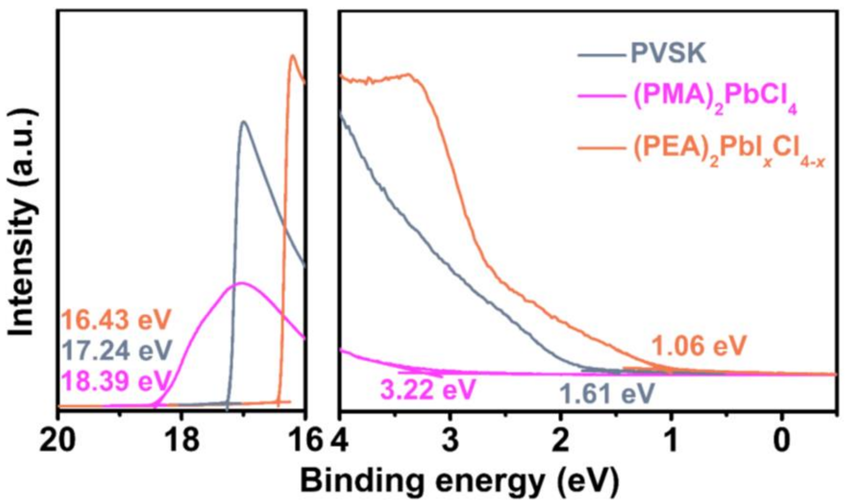

Fig. S14 UPS spectra of the control film, 2D $(PMA)_2PbCl_4$, and 2D $(PEA)_2PbI_xCl_{4-x}$

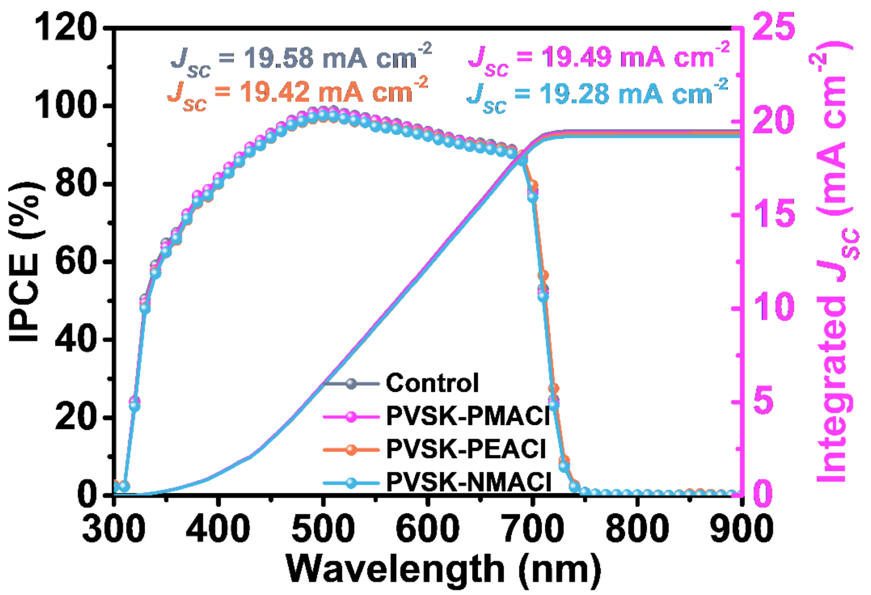

Fig. S15 IPCE and the integrated J_{SC} for the WBG-PSCs based on the control, PVSK-PMACl, PVSK-PEACl, and PVSK-NMACl films

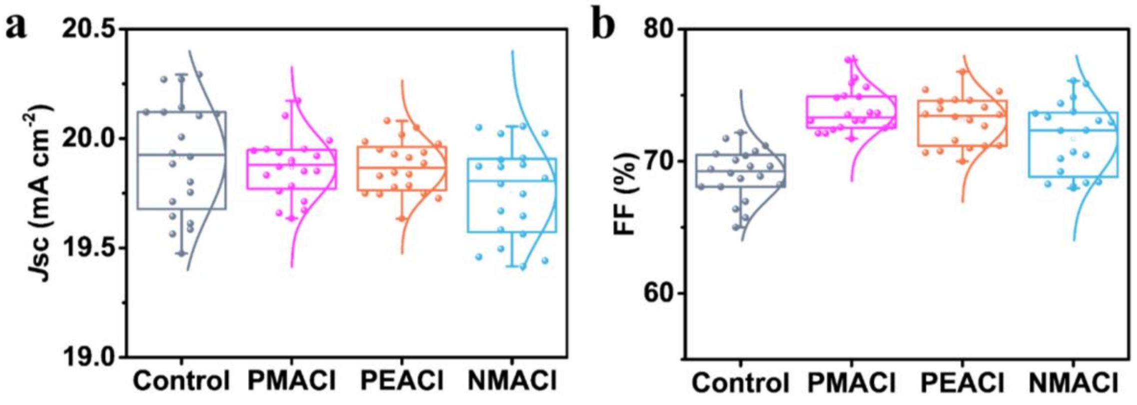

Fig. S16 Statistical results of **a** J_{SC} and **b** FF of 20 PSCs for each group

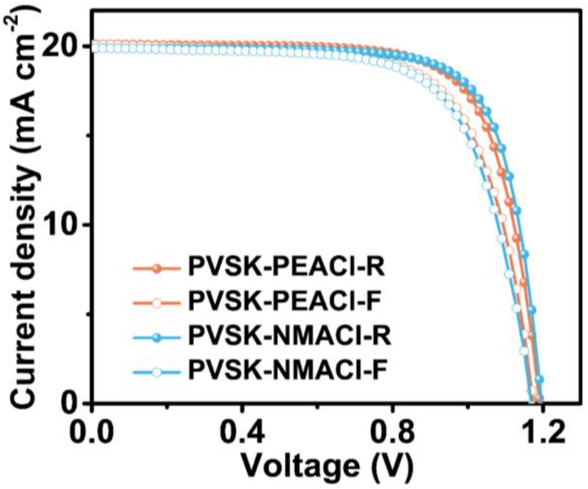

Fig. S17 *J-V* curves of the WBG-PSCs based on the PVSK-PEACl, and PVSK-NMACl films measured by forward and reverse scans

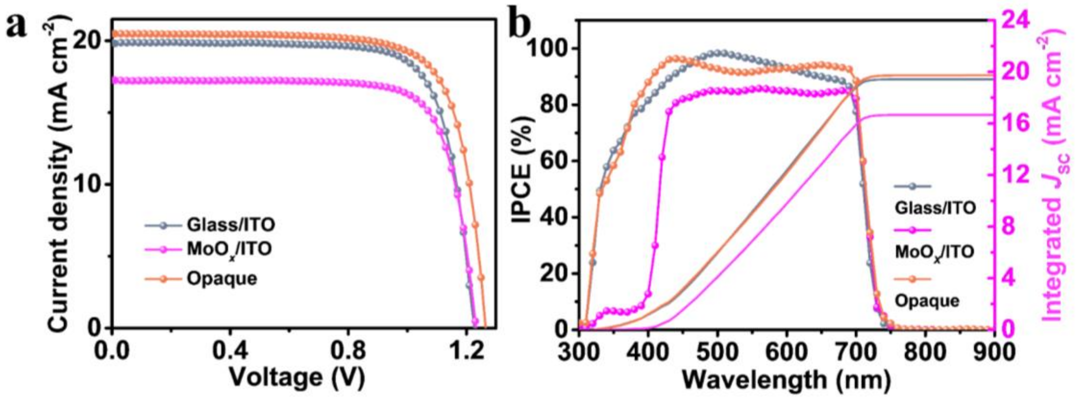

Fig. S18 **a** *J-V* curves and **b** IPCE spectra of solar cells. Glass/ITO represents light incident from the glass/ITO side of the semi-transparent cell, MoOx/ITO represents light incident from the MoOx/ITO side of the semi-transparent cell, and opaque represents the device using Ag electrode

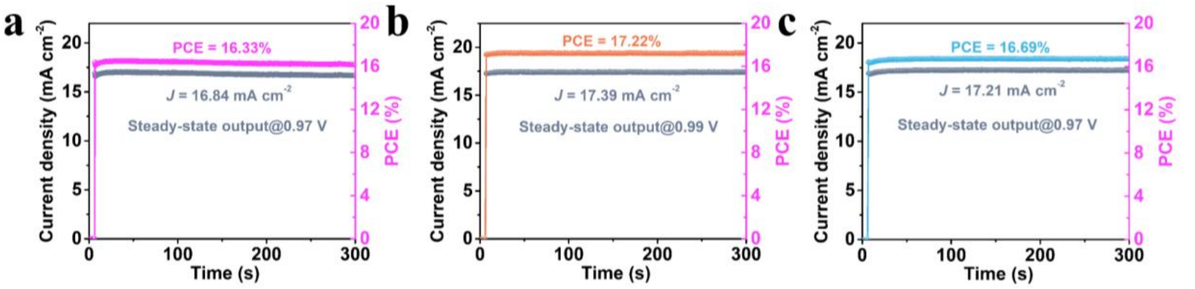

Fig. S19 Maximum power point tracking of the WBG-PSCs based on the **a** control, **b** PVSK-PEACl, and **c** PVSK-NMACl films, respectively

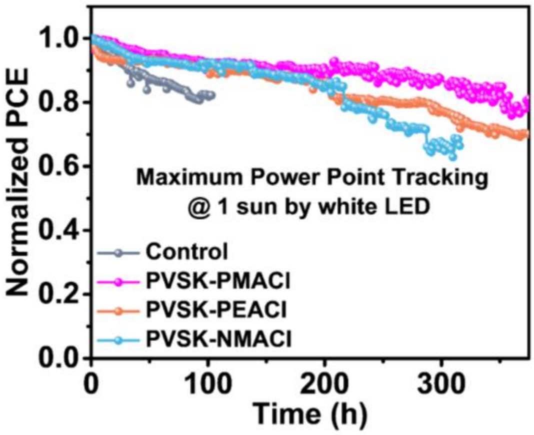

Fig. S20 Maximum power point tracking under white LED lamp with the light intensity of 100 mW cm^{-2}

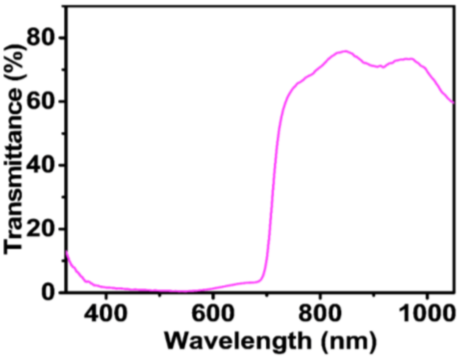

Fig. S21 Transmittance spectrum of semi-transparent WBG-PSCs

Table S1 XRD peak intensity of 2D $(PMA)_2PbI_xCl_{4-x}$ annealed at different temperatures

T/℃	40	60	80	90	100	110	120
5.32	5933	9481	20066	23878	28353	19650	11635
6.20	8367	45474	148273	154726	139958	72485	3311
5.32/6.20	0.71	0.21	0.14	0.15	0.20	0.27	3.5

Table S2 Assignment of the XRD peaks of different 2D perovskites

	(001)	(002)	(003)	(004)	(005)	(006)
$(PMA)_2PbI_4$	6.20	12.34	18.54	24.80	31.12	37.54
$(PMA)_2PbCl_4$	5.32	10.56	15.84	21.14	26.52	31.94
$(PMA)_2Pb(I_{1-x}Cl_x)_4$	5.32/ 6.20	10.56/ 12.34	15.84 /18.54	21.14/ 24.80	26.52/ 31.12	31.94/ 37.54
$(PEA)_2PbI_4$	5.46	10.84	16.28	21.74	27.26	32.86
$(PEA)_2PbCl_4$	5.28	10.50	15.74	21.04	26.38	31.76
$(PEA)_2Pb(I_{1-x}Cl_x)_4$	5.22	10.40	15.58	20.80	26.06	31.40

Table S3 Fitted parameters of TRPL results of the control, PVSK-PMACl, PVSK-PEACl, and PVSK-NMACl films

Samples	τ_1/ns	A_1	τ_2/ns	A_2	τ_{avg} (ns)
Control	79.3	0.7	353.2	0.8	309.5
PVSK-PMACl	167.6	0.3	962.9	0.8	914.1
PVSK-PEACl	111.4	0.9	816.6	0.5	675.9
PVSK-NMACl	98.1	1.1	474.4	0.6	369.4

Table S4 Photovoltaic parameters of the devices based on the control, PVSK-PMACl, PVSK-PEACl, and PVSK-NMACl films measured by forward and reverse scans

Samples	Scanning direction	J_{SC} (mA cm^{-2})	V_{OC} (V)	FF (%)	PCE (%)	HI (%)
Control	Reverse	19.71	1.181	70.98	16.52	15.25
	Forward	19.70	1.163	61.09	14.00	
PVSK-PMACl	Reverse	19.78	1.224	75.91	18.38	4.19
	Forward	19.75	1.207	73.82	17.62	
PVSK-PEACl	Reverse	20.10	1.191	73.13	17.51	6.85
	Forward	20.09	1.174	69.17	16.31	
PVSK-NMACl	Reverse	19.91	1.196	74.86	17.82	9.99
	Forward	19.91	1.169	68.92	16.04	

Table S5 Champion photovoltaic parameters of solar cells. Glass/ITO represents light incident from the glass/ITO side of the semi-transparent cell, MoO_x/ITO represents light incident from the MoO_x/ITO side of the semi-transparent cell, and opaque represents the device using Ag electrode

Samples	J_{SC} (mA cm^{-2})	V_{OC} (V)	FF (%)	PCE (%)
Glass/ITO	19.87	1.23	76.31	18.60
MoO_x/ITO	17.26	1.23	77.19	16.42
Opaque	20.49	1.27	75.68	19.62
